# Structural basis and selectivity of sulfatinib binding to FGFR and CSF-1R

**DOI:** 10.1038/s42004-023-01084-0

**Published:** 2024-01-03

**Authors:** Qianmeng Lin, Shuyan Dai, Lingzhi Qu, Hang Lin, Ming Guo, Hudie Wei, Yongheng Chen, Xiaojuan Chen

**Affiliations:** 1 Department of Oncology, NHC Key Laboratory of Cancer Proteomics, State Local Joint Engineering Laboratory for Anticancer Drugs, Xiangya Hospital, Central South University, Changsha, Hunan 410008 China; 2 National Clinical Research Center for Geriatric Disorders, Xiangya Hospital, Central South University, Changsha, Hunan 410008 China

**Keywords:** X-ray crystallography, Kinases, Structure-based drug design, Structure-based drug design

## Abstract

Acquired drug resistance poses a challenge for single-target FGFR inhibitors, leading to the development of dual- or multi-target FGFR inhibitors. Sulfatinib is a multi-target kinase inhibitor for treating neuroendocrine tumors, selectively targeting FGFR1/CSF-1R. To elucidate the molecular mechanisms behind its binding and kinase selectivity, we determined the crystal structures of sulfatinib with FGFR1/CSF-1R. The results reveal common structural features and distinct conformational adaptability of sulfatinib in response to FGFR1/CSF-1R binding. Further biochemical and structural analyses disclose sensitivity of sulfatinib to FGFR/CSF-1R gatekeeper mutations. The insensitivity of sulfatinib to FGFR gatekeeper mutations highlights the indispensable interactions with the hydrophobic pocket for FGFR selectivity, whereas the rotatory flexibility may enable sulfatinib to overcome CSF-1R^T663I^. This study not only sheds light on the structural basis governing sulfatinib’s FGFR/CSF-1R inhibition, but also provides valuable insights into the rational design of dual- or multi-target FGFR inhibitors with selectivity for CSF-1R and sensitivity to gatekeeper mutations.

## Abstract (web summary)

Sulfatinib is a multi-target angio-immuno kinase inhibitor for treating neuroendocrine tumors, selectively targeting FGFR1 and CSF-1R. Here, the authors elucidate the molecular mechanisms behind its binding and kinase selectivity, highlighting interactions with a hydrophobic pocket for FGFR selectivity, and rotatory flexibility to potentially overcome CSF-1R^T663I^ gatekeeper mutation.

## Introduction

The fibroblast growth factor (FGF)-FGF receptor (FGFR) axis plays vital roles in angiogenesis, endocrine functions, wound repair as well as tissue and metabolism homeostasis^1–3^. It regulates multiple downstream signaling pathways, including RAS–RAF–MAPK, PI3K–AKT, signal transducer and activator of transcription (STAT) and phospholipase Cγ (PLCγ), to control cellular proliferation, differentiation, survival, and migration^3–5^. Recently, FGFR gene amplification, activating mutations, and gene rearrangements or fusions, have been documented abundantly in many tumor types, such as cholangiocarcinoma, urothelial carcinoma, and gastric cancer^2,6,7^. These alterations in FGF/FGFR signaling are closely related to tumorigenesis, tumor progression, and drug resistance, rendering FGFR as a potential target for cancer therapy^2,4^.

Numerous small-molecule FGFR inhibitors have been clinically approved or have become candidate drugs for cancer therapy. Unfortunately, acquired drug resistance gradually develops after an initial response to these molecules through both “on-target” and “off-target” mechanisms^1,8,9^. To address the on-target resistance, higher drug dosages are often required to ensure adequate accessibility to tumor tissues, resulting in non-specific targeting and dose-limited toxicity^8–10^. On the other hand, combination therapy is employed to overcome the off-target resistance but increases the risk of drug-drug interaction^8,9^. Therefore, the development of dual- or multi-target FGFR inhibitors has emerged to improve therapeutic effects and patient compliance, while reducing risks of drug-drug interactions and acquired resistance associated with single-target FGFR inhibitors. However, due to their poor selectivity, these compounds are always related to off-target effects and adverse events, significantly impeding their clinical applications^9^. Consequently, there is a pressing need to develop dual- or multi-target FGFR inhibitors with high selectivity and potent inhibitory activity.

**Fig. 1:**
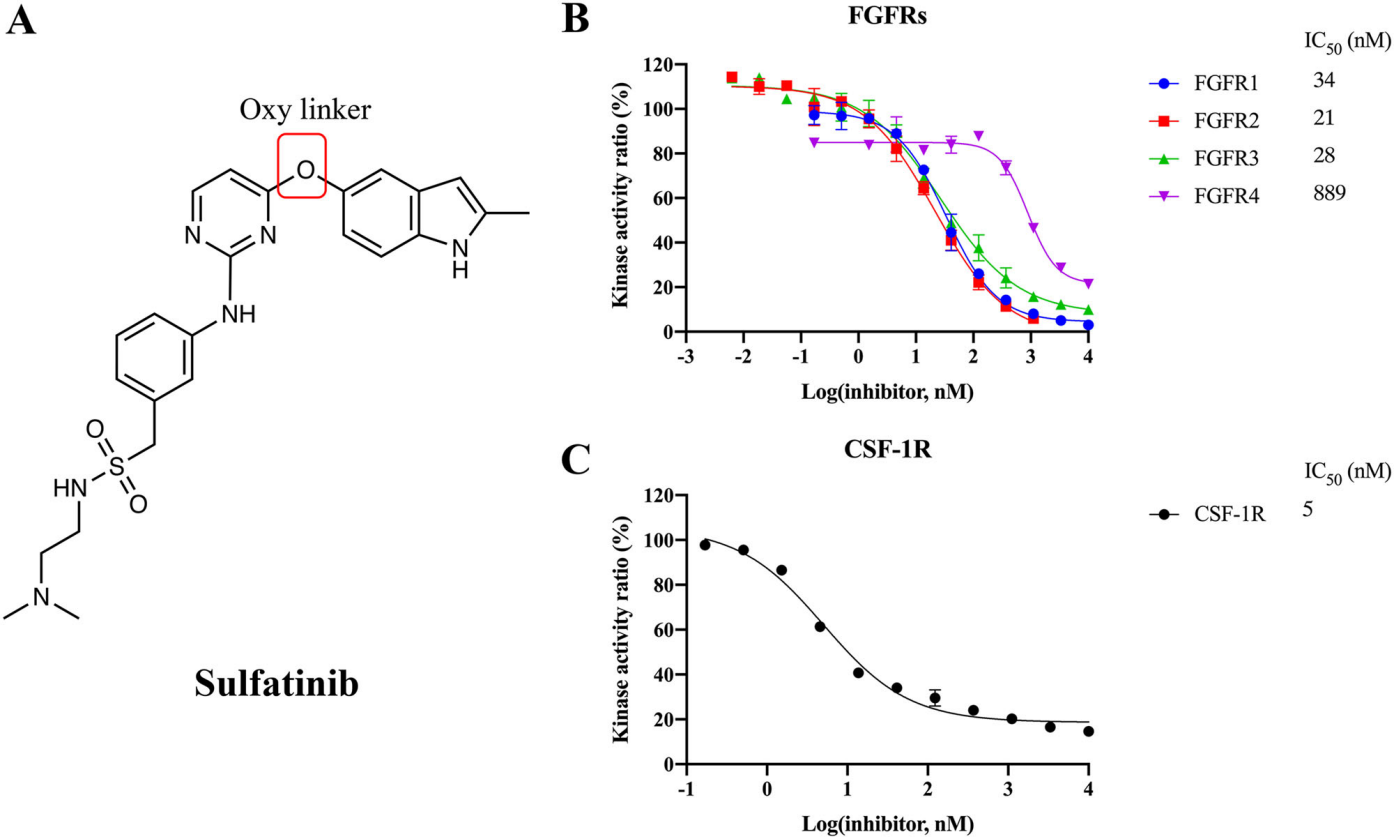
Sulfatinib exhibits great potency against FGFR1-3 and CSF-1R. **A** Chemical structures of sulfatinib. Inhibitory effects of sulfatinib on wild-type FGFR1-4 **B** and CSF-1R **C** through kinase activity inhibition assays. Error bars represent the standard deviation for at least three independent measurements.

Sulfatinib (Fig. 1A) is a multi-target drug that selectively targets FGFR, collection stimulating factor 1 receptor (CSF-1R), and vascular endothelial growth factor receptor (VEGFR). It has been approved for the treatment of pancreatic and extrapancreatic neuroendocrine tumors (NETs)^11–14^. CSF-1R can induce the proliferation, survival, and differentiation of tumor-associated macrophages, which are abundant in various tumor-microenvironment activities, thereby promoting tumor immune evasion and drug resistance^15–17^. Blockade of CSF-1R signaling can reshape the tumor microenvironment and enhance immune responses, making CSF-1R an attractive target combined with FGFR for cancer therapy. Additionally, VEGFR is up-regulated with FGFR genetic changes, promoting tumor angiogenesis that is essential for tumor growth, invasion, and metastasis^18,19^. Therefore, the encouraging antitumor activity of sulfatinib for NETs is achieved by preventing tumor angiogenesis and tumor immune evasion through simultaneous FGFR, CSF-1R, and VEGFR inhibition^11–14^. Sulfatinib exhibits great potency and selectivity for these kinases, with minimal off-target effects^8,9,11–14^, making it an ideal template for developing FGFR inhibitors with dual- or multi-targeting capabilities. Thus, understanding how sulfatinib simultaneously targets these kinases is of utmost importance.

In this study, we aimed to elucidate the structural basis underlying the high potency and selectivity of sulfatinib against FGFR and CSF-1R. To achieve this, we determined the crystal structures of sulfatinib in complex with FGFR1 and CSF-1R using X-ray crystallography. We conducted structural comparisons of sulfatinib bound to different kinases and with other specific inhibitors to reveal common structural features and distinct conformational adaptability of sulfatinib in response to different kinase binding pockets. Furthermore, we evaluated the resistance profiles and mechanisms of sulfatinib inhibition against FGFR and CSF-1R gatekeeper mutations through biochemical and structural analysis. Collectively, our work not only sheds light on the molecular mechanisms of the dual inhibition of FGFR and CSF-1R conferred by sulfatinib, but also provides fundamental information for the rational design of dual- or multi-target FGFR inhibitors with high selectivity for CSF-1R and sensitivity to gatekeeper mutations.

## Results

### Potent inhibition of FGFRs and CSF-1R by sulfatinib

A kinase inhibition assay was employed to confirm the inhibitory potency of sulfatinib against FGFRs and CSF-1R. Consistent with the previous reports^20^, sulfatinib demonstrated potent inhibition of FGFR1 and CSF-1R, with IC_50_ values of 34 and 5 nM, respectively (Fig. 1B, C). The high sequence homology among the FGFRs family indicates that sulfatinib may exhibit similar efficacy against other members. As anticipated, sulfatinib effectively inhibited FGFR2 and FGFR3, comparable to FGFR1, with low nanomolar IC_50_ values (21 nM and 28 nM, respectively), whereas a reduction in efficacy of approximately 26-fold was observed for FGFR4 (IC_50_ value of 889 nM). These findings establish sulfatinib as a potent inhibitor of FGFR1-3 and CSF-1R.

### Structural basis of the sulfatinib/FGFR1 interaction

**Fig. 2:**
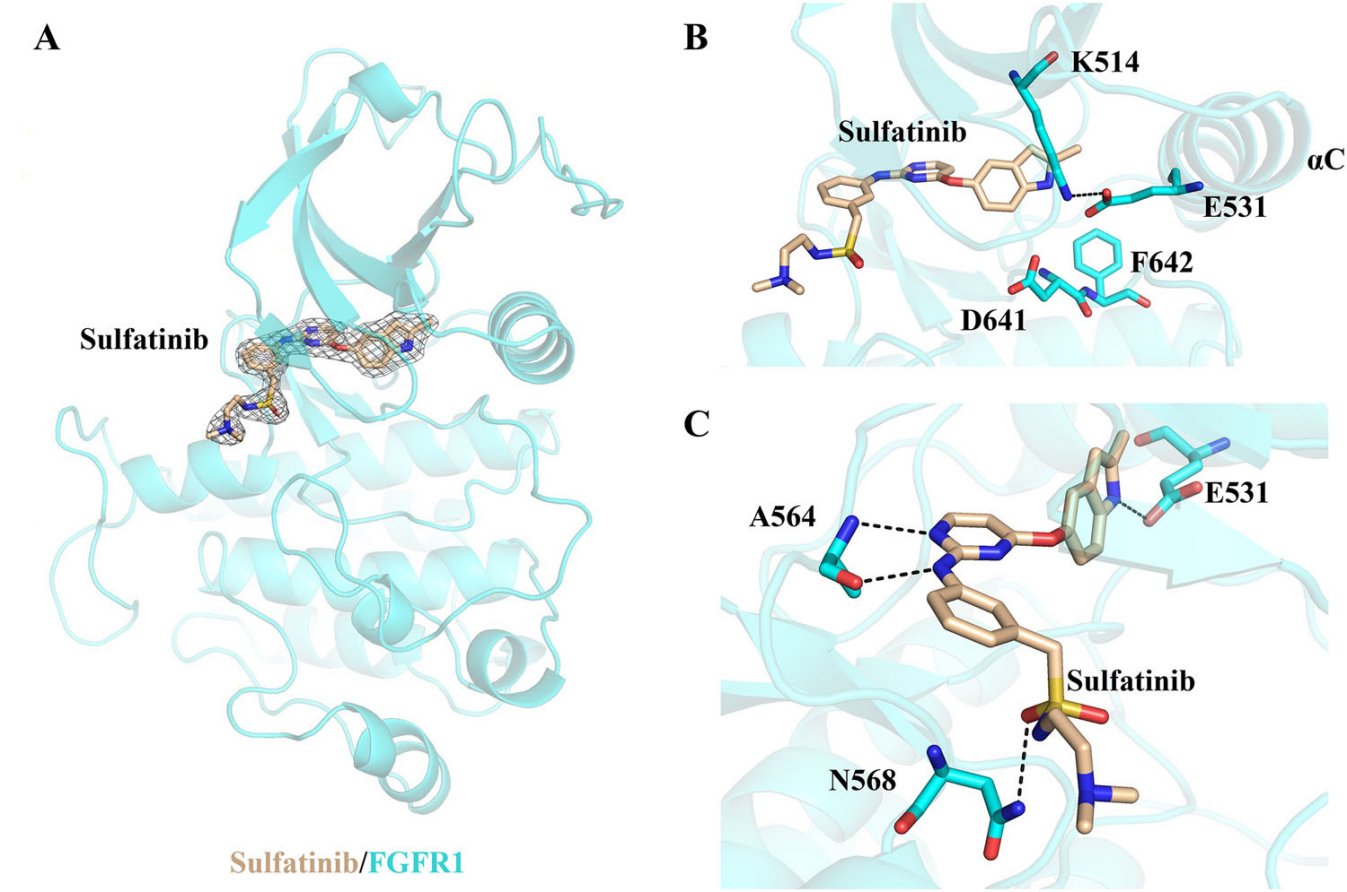
Crystal structure of sulfatinib in complex with FGFR1. **A** Overall structure of the sulfatinib/FGFR1 complex. **B** FGFR1 adopts a DFG-in activation loop conformation. **C** Hydrogen-bond interaction between sulfatinib and FGFR1.

To gain structural insights into the mechanism of FGFR inhibition by sulfatinib, we solved the crystal structure of sulfatinib with FGFR1 at 1.988 Å resolution (PDB 8JMZ) (Supplementary Data 1). Data collection and structure refinement statistics are presented in Supplementary Table 1. The overall structure of sulfatinib/FGFR1 complex is described in Fig. 2A, with the activation loop adopting a DFG-in conformation (Fig. 2B). As illustrated in Fig. 2C, the pyrimidine nitrogen and the adjacent phenylamine NH of sulfatinib make a bidentate hydrogen-bonding interaction with Ala564 at the hinge region of FGFR1, whereas the indole group fills the hydrophobic pocket and forms a hydrogen bond with Glu531. The N-(2-dimethylamino-ethyl)-ethanesulfonamide group is exposed to the solvent-exposed region and makes a hydrogen-bond interaction with Asn568. This binding mode is also observed in the FGFR1/infigratinib complex (PDB 3TT0, Supplementary Fig. 1), in which infigratinib is a selective and potent FGFR1-3 inhibitor^21^. Overall, sulfatinib binds to a DFG-in conformation of FGFR1 by the extensive interaction network in the ATP pocket, corresponding to a type I inhibitor.

### Structural insight into the sulfatinib/CSF-1R interaction

**Fig. 3:**
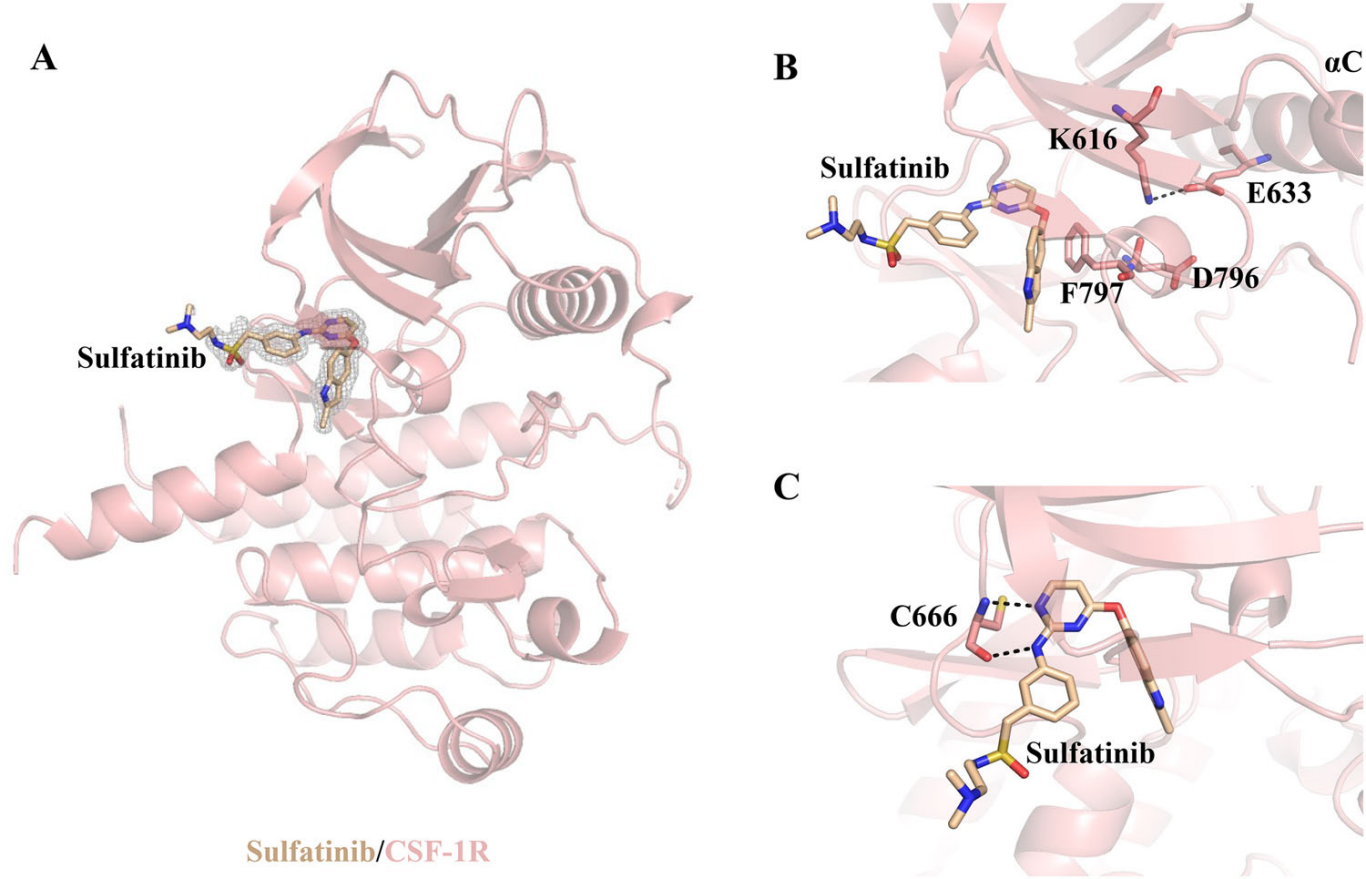
Crystal structure of sulfatinib in complex with CSF-1R. **A** Overall structure of the sulfatinib/CSF-1R complex. **B** CSF-1R adopts a DFG-out activation loop conformation. **C** Hydrogen-bond interaction between sulfatinib and CSF-1R.

To provide a foundation for understanding the mechanism that enables sulfatinib to govern FGFRs and CSF-1R simultaneously, we then solved the cocrystal structure of CSF-1R with sulfatinib at 1.69 Å resolution (PDB 8JOT) (Supplementary Data 2). Data collection and structure refinement statistics are presented in Supplementary Table 1. The overall CSF-1R/sulfatinib binding pattern is well-presented in Fig. 3A. Specifically, CSF-1R adopts a DFG-out activation loop upon sulfatinib binding (Fig. 3B). Sulfatinib maps in the ATP-binding pocket of CSF-1R with the pyrimidine nitrogen and the adjacent phenylamine NH making a bidentate hydrogen bond with the hinge residue Cys666 and the N-(2-dimethylamino-ethyl)-ethanesulfonamide moiety extending toward the solvent-exposed area (Fig. 3C). Unexpectedly, the indole group of sulfatinib does not occupy the hydrophobic pocket but rotates out in response to the DFG-out flip. These results demonstrate that sulfatinib inhibits CSF-1R by locking the protein in an inactive state, classified as a type II inhibitor.

### Structural comparison of sulfatinib in complex with FGFR1 and CSF-1R

**Fig. 4:**
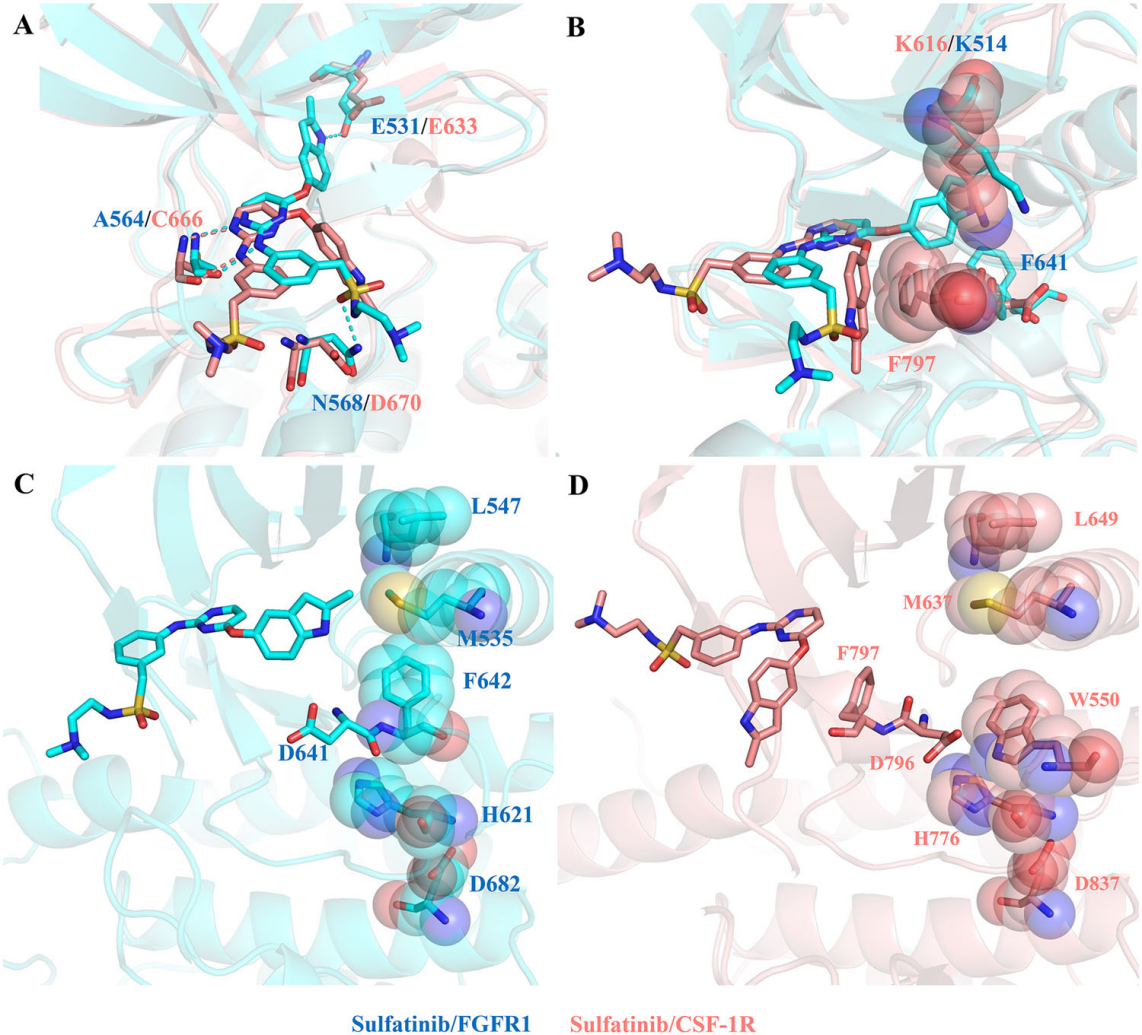
Structural comparison of sulfatinib in complex with FGFR1 and CSF-1R. **A** Comparison of sulfatinib/FGFR1 interaction and sulfatinib/CSF-1R interaction. **B** FGFR1-bound sulfatinib may clash with Lys616 and Phe797 of CSF-1R when overlaying the sulfatinib/FGFR1 complex and sulfatinib/CSF-1R complex. **C** The R-spine of FGFR1 is linear and consists of Leu547, Met535, Phe642, His621 and Asp682. **D** The R-spine of CSF-1R is broken by the substitution of Phe797 with Trp550 from the juxtamembrane domain, which enforces the displacement of Phe797 to the DFG-out conformation. FGFR1 is colored cyan, while CSF-1R is colored salmon.

The kinase domains of FGFR1 and CSF-1R share significant sequence similarity, especially the residues around the ligand-binding pocket^22^. However, the binding modes of these two kinases bound to sulfatinib are extremely distinct. To figure out the structural basis for the different inhibitory mechanisms, we superposed the two complexes (Fig. 4A). Sulfatinib exhibits dramatic conformational changes upon FGFR1 and CSF-1R except for the hinge-interacting pattern. The indole group of sulfatinib in CSF-1R is flipped out of the hydrophobic pocket via an oxy linker. In contrast, the solubilizing group is rotated by 180° in proximity to the hinge region of CSF-1R, compared with that of FGFR1. These conformational differences in sulfatinib lead to the distinct interaction contacts between FGFR1 and CSF-1R (Supplementary Fig. 2). We supposed that sulfatinib may bind to CSF-1R as the mode with FGFR1, but the indole group of sulfatinib may clash with Lys616 and Phe797 of CSF-1R (Fig. 4B), which means it may have a higher-energy barrier to attain the FGFR1-sulfatinib binding mode (Supplementary Fig. 3A). Thus, the indole group of sulfatinib in CSF-1R rearranges to a less energetically favorable conformation (Supplementary Fig. 3B).

Apart from the discrepancies in sulfatinib conformation, there is a distinguished conformational difference between FGFR1 and CSF-1R, as the Asp and Phe side chains of the DFG motif swap positions. In the FGFR1 structure, Phe642 is involved in the linear R-spine of FGFR1 that is made up of Leu547, Met535, Phe642, His621, and Asp682 (Fig. 4C), which is a hallmark signature of the active kinase state^23,24^. As to CSF-1R, the R-spine is broken and Phe797 is displaced by Trp550 from the juxtamembrane domain (JMD) (Fig. 4D), thereby forcing the DFG-out flip and forming an inhibitory R-spine. These results indicate that these conformational alterations are associated with the Asp-Phe swap, showing the flexibility and adaptability of sulfatinib upon different kinase bindings.

### Structural comparison of CSF-1R in complex with sulfatinib and vimseltinib

**Fig. 5:**
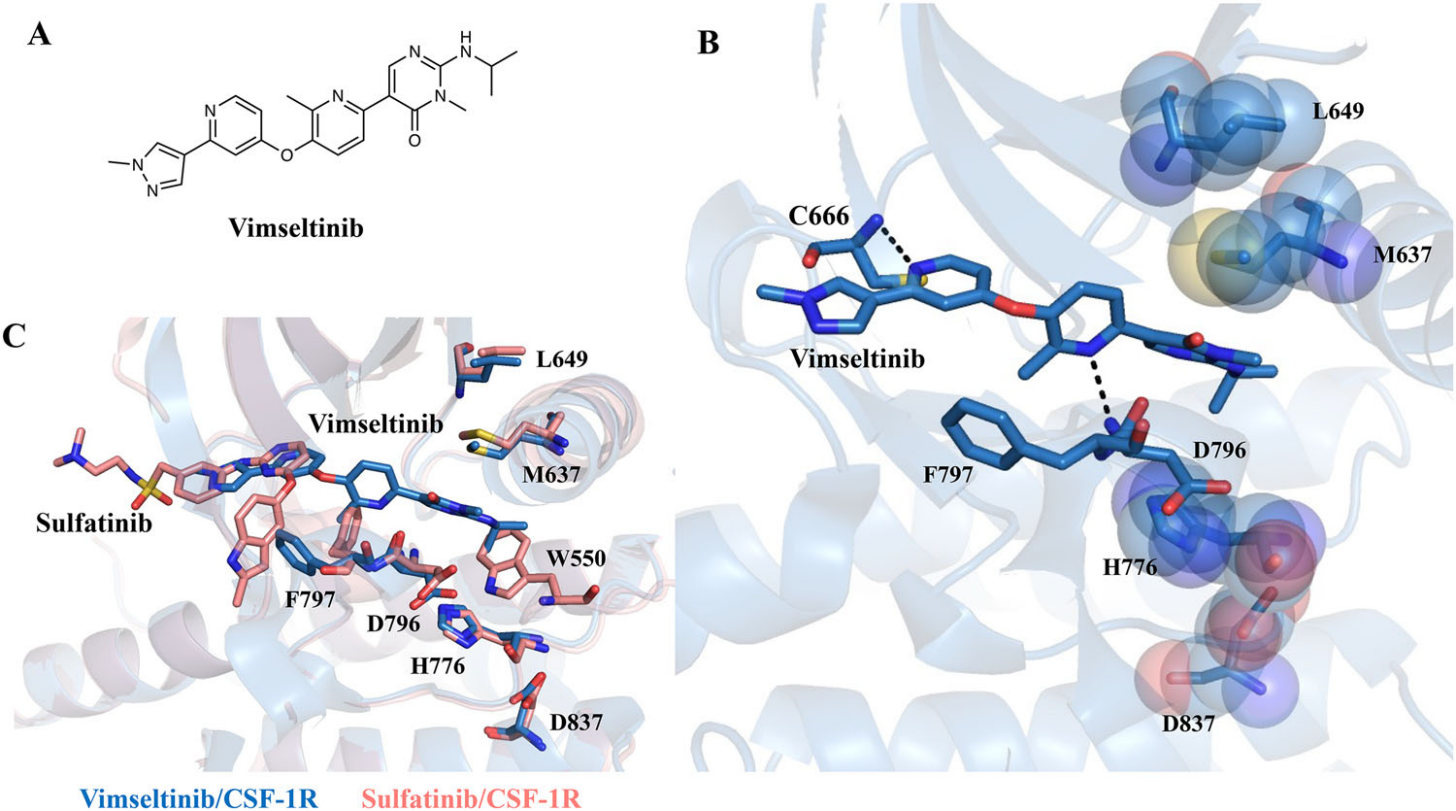
Structural comparison of CSF-1R bound by vimseltinib and sulfatinib. **A** Chemical structure of vimseltinib. **B** Binding mode of vimseltinib in complex with CSF-1R (PDB 7MFC). **C** Superposition of sulfatinib/CSF-1R (salmon) and vimseltinib/CSF-1R (sky blue).

To explore the structural features in ligand binding by the same receptor, we compared the structures of CSF-1R with sulfatinib and vimseltinib (PDB 7MFC), a potent and selective CSF-1R inhibitor (Fig. 5A). Vimseltinib has a similar hydrogen-binding interaction pattern with the hinge residue as sulfatinib^25^ (Fig. 5B). The indole substituent of sulfatinib and the methylpyridine ring of vimseltinib are both connected by an oxy linker to the hinge-interacting group but present a completely opposite conformation (Fig. 5C). The indole substituent of sulfatinib is flipped out, while the methylpyridine ring coupled with the N-isopropyl pyrimidinone group extends deeply into the hydrophobic pocket, serving as a surrogate for the JMD Trp550 and stabilizing the hydrophobic R-spine. To better accommodate vimseltinib, the Phe797 residue in turn slightly spins out to avoid steric clash. Moreover, the methylpyridine ring of vimseltinib interacts with Asp796 of the DFG motif, which helps vimseltinib firmly anchored in this pocket. These results suggest that the selectivity for CSF-1R could be improved by the occupation of the third position of the R-spine and the hydrogen binding with Asp796.

### Efficacy of sulfatinib in response to FGFRs and CSF-1R gatekeeper mutations

**Fig. 6:**
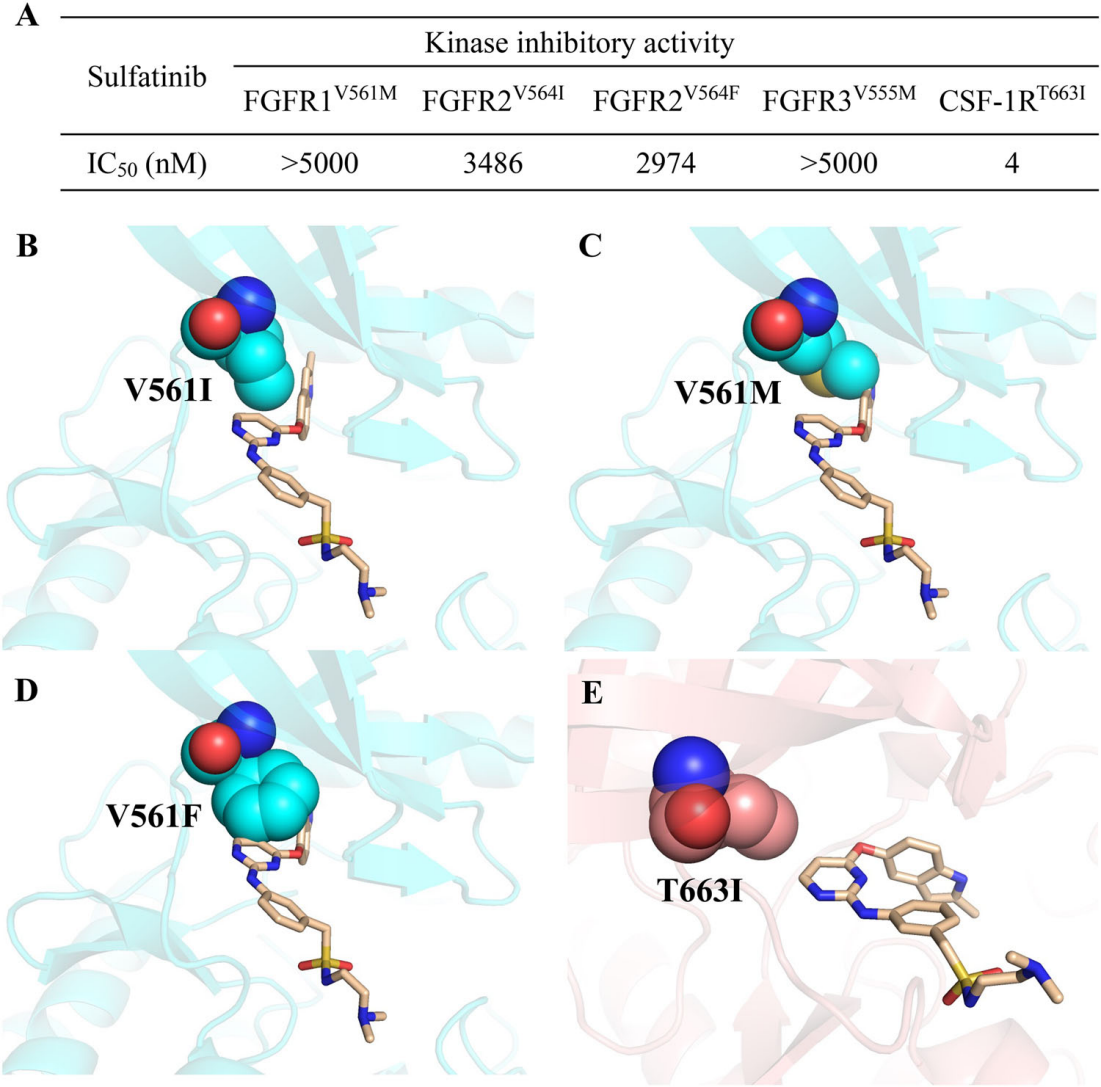
Sensitivity of sulfatinib to FGFR and CSF-1R gatekeeper mutations. **A** Inhibitory effects of sulfatinib on FGFR and CSF-1R gatekeeper mutations through kinase activity inhibition assays. **B**–**E** Structural models of sulfatinib in complex with FGFR1^V561I/M/F^ and CSF-1R^T663I^, which are generated by substitution of gatekeeper residues based on our solved FGFR1/sulfatinib (PDB 8JMZ) and CSF-1R/sulfatinib (PDB 8JOT) structures. Error bars represent the standard deviation for at least three independent measurements.

The “gatekeeper” residue of protein kinase controls the access of kinase inhibitors to the ATP-binding pocket, by which mutations to a larger hydrophobic residue are responsible for drug resistance. Therefore, it is crucial to counter the problem of the sensitivity of sulfatinib to FGFRs and CSF-1R gatekeeper mutations. Kinase activity inhibition assays were carried out. Unfortunately, sulfatinib cannot overcome any FGFR gatekeeper mutation with IC_50_ values all over 2 μM for FGFR1^V561M^, FGFR2^V564I/F^ and FGFR3^V555M^ (Fig. 6A). In order to explain this on-target resistance mechanism, we constructed the binding models of FGFR1^V561I/M/F^ in complex with sulfatinib based on our solved FGFR1/sulfatinib complex (Fig. 6B–D). These structural models predict that the bulkier side chains of isoleucine, methionine and phenylalanine residues may clash with sulfatinib and prevent the extension of sulfatinib into the hydrophobic pocket, thereby leading to the observed loss in potency.

All FGFR kinases possess a valine gatekeeper, in contrast to CSF-1R, c-Kit, PDGFRα and BCR-ABL, which all have a threonine at the gatekeeper position (Supplementary Fig. 4). Although there are no mutations reported upon Thr663 gatekeeper of CSF-1R, c-Kit^T670I^, PDGFRα^T674I^ and BCR-ABL^T315I^ have been found causally linked to drug resistance^26^, which means that CSF-1R would develop T663I gatekeeper mutation during the clinical usage. The kinase activity inhibition assay results showed that sulfatinib preserved a remarkable inhibitory effect on CSF-1R^T663I^ with IC_50_ value of 4 nM (Fig. 6A). Here, we proposed the binding model of CSF-1R^T663I^ with sulfaitnib based on our solved CSF-1R/sulfatinib structure. It seems that there is a large space available to accommodate sulfatinib without steric clash with the isoleucine side chain (Fig. 6E), supporting the high sustainability of sulfatinib against CSF-1R^T663I^ mutation as determined by biochemical assay. Collectively, these structural models suggest that sulfatinib cannot overcome FGFR gatekeeper mutations due to the severe hindrance from the bulky isoleucine, methionine and phenylalanine side chains, but exhibit persistent activity against CSF-1R^T663I^ attributing to the distinct conformational adaptation of sulfatinib upon CSF-1R binding.

## Discussion

Sulfatinib stands out as a potent and selective multi-target inhibitor, with a lower rate of off-target side effects compared to other reported dual- or multi-target FGFR inhibitors, such as lenvatinib, sunitinib, pazopanib, and nintedanib. This can be attributed to sulfatinib’s high potency and selectivity for FGFR, CSF-1R, and VEGFR over 278 other kinases^9,20,27–29^. As anticipated, we confirmed that sulfatinib has significant potency against FGFR1-3 and CSF-1R with low nanomolar IC_50_ values (Fig. 1B, C). This finding suggests that sulfatinib has the potential for therapeutic applicability in FGFR-driven malignancies characterized by prominent immune evasion profiles. Tumor immunotherapy remains a highly attractive but not yet fully operational treatment approach^30^. To date, the FDA has approved thirty-one combinations of tyrosine kinase inhibitors with immunotherapy for various indications, and numerous ongoing clinical trials continue to explore the combinations, reflecting an ever-increasing interest in incorporating immunosurveillance into kinase inhibitors^31^. Given the significant role of CSF-1R in tumor immune modulation, our focus was primarily on the roles of FGFR and CSF-1R in the context of sulfatinib.

To elucidate the underlying mechanism of inhibitor binding and kinase selectivity for sulfatinib, we solved crystal structures of sulfatinib in complex with FGFR1 and CSF-1R by X-ray crystallization. The N-phenyl-2-pyrimidinamine core scaffold of sulfatinib, forming hydrogen-bound interactions with the hinge region, proves essential for the inhibitory effects on FGFR and CSF-1R, which may serve as an optimal template for the rational design of FGFR inhibitors with CSF-1R selectivity. Notably, the flexibility provided by the oxy linker of sulfatinib emerges as a critical factor for the FGFR and CSF-1R targeting, allowing sulfatinib to adapt and adopt multiple binding conformations in response to different kinase bindings. Furthermore, the occupancy of the indole group of sulfatinib within the hydrophobic pocket appears to enhance the selectivity for FGFR. Introducing bulky isoleucine, methionine, and phenylalanine residues at the gatekeeper position of FGFR leads to severe steric clash with the indole group of sulfatinib. Consequently, the clash disrupts the hydrogen binding with Ala564 of the hinge region and Glu531 surrounding the hydrophobic pocket^32–34^. The flexible oxy linker may assist in shifting the indole group out of this pocket, resulting in a CSF-1R/sulfatinib-like binding model to better accommodate gatekeeper mutation and maintain hydrogen contacts with the hinge residue. However, this conformational change of sulfatinib may lead to a complete loss of inhibitory activity against FGFR, as evidenced by the micromolar range of IC_50_ values for FGFR1^V561M^, FGFR2^V564I/F^, and FGFR3^V555M^ (Fig. 6A). This emphasizes the critical role of the occupancy at the hydrophobic pocket for FGFR selectivity.

Apart from sulfatinib, PRN1371 also had great potency and selectivity against FGFR1-4 and CSF-1R with IC_50_ values of 0.6, 1.3, 4.1, 19.3, and 8.1 nM, respectively^35^. We compared the FGFR1/sulfatinib complex with FGFR4/PRN1371 complex (PDB 7F3M)^6^, and found that these two binding modes are similar, with hydrogen-bound interactions with the hinge region and occupancy of hydrophobic pocket via a hydrogen bond (Supplementary Fig. 5A, B). The bulkier difluoromethoxyphenyl ring of PRN1371 than the indole group of sulfatinib may also clash with bulky isoleucine, methionine, and phenylalanine residues at the gatekeeper position, inducing insensitivity to FGFR gatekeeper mutations. As for CSF-1R binding, we predicted the binding mode of PRN1371 with CSF-1R, and made a comparison with our crystal of sulfatinib/CSF-1R (Supplementary Fig. 5C, D). CSF-1R kept a DFG-out conformation when binding to sulfatinib but tended to adopt a DFG-in conformation in response to PRN1371 binding, in which the latter Asp of DFG forming a hydrogen bond with the difluoromethoxyphenyl ring of PRN1371 firmly anchored this ring into the hydrophobic pocket. In contrast to PRN1371, the structural flexibility of sulfatinib allowed the indole flip out of the pocket even to escape the clash with the bulky gatekeeper residues, which was absolutely not allowed for PRN1371 due to its structural rigidity. Collectively, the binding mode comparison of the two inhibitors bound by FGFR/CSF-1R highlighted the common structural features and the distinct conformational adaptability in the inhibitor-kinase interactions.

Our structure of CSF-1R/FGFR1 with sulfatinib offers significant opportunities for developing dual- or multi-target FGFR inhibitors with improved potency against CSF-1R. For example, the close proximity of the side chain of Asp796 in CSF-1R to the indole group of sulfatinib when occupying the hydrophobic pocket suggests the possibility of hydrogen-bond interactions after proper modifications to anchor this pharmacophore. This observation aligns with the designs of several selective CSF-1R inhibitor^36–41^. Moreover, the residues around the hydrophobic pocket exposed in the DFG-out conformation of CSF-1R show less conserved across kinases, suggesting the possibility of achieving high selectivity for CSF-1R^42^. Inspired by the structural comparison of CSF-1R with sulfatinib and vimseltinib^36,37^, incorporating a surrogate for JMD Trp550 or the formation of hydrophobic interactions with Trp550 may stabilize the hydrophobic R-spine and reinforce sulfatinib binding. Currently, pharmacophore-based combination strategies are widely applied in drug design^8^, which indicates that combining distinct pharmacophores from sulfatinib and vimseltinib or other selective CSF-1R inhibitors is conducive to the rational design of dual- or multi-target FGFR inhibitors with CSF-1R selectivity to improve antitumor efficiency.

To prolong the therapeutic efficacy, further development to overcome gatekeeper mutation-based resistance is warranted. Based on the crystal of FGFR1/CSF-1R with sulfatinib, there are some strategies to preserve sustainable inhibitions against FGFR/CSF-1R gatekeeper mutations. The indole group of sulfatinib could be substituted with other heterocyclic fragments by hydrogen-bond interactions with Asp of DFG motif to move far away from gatekeeper residues, or with other smaller moieties to provide enough space for gatekeeper residues, which may not only avoid steric clash but also meet the requirement of occupying the hydrophobic pocket for FGFR selectivity. Introducing a slim and long linker, such as the alkyne linker in ponatinib^43^, into the inhibitor core scaffold may also allow avoidance of steric clash and greater spacing to better accommodate a bulky residue at the gatekeeper position. Encouraged by the flexible oxy linker in sulfatinib, exploiting inhibitor flexibility by incorporation of a flexible linker, such as the ethyl linker in AZD4547^32^, may be an effective strategy to curb drug resistance by allowing diverse inhibitor binding modes.

In conclusion, we confirmed that sulfatinib has great potency against FGFR1-3 and CSF-1R by kinase inhibition assay. The structural analysis and comparisons have provided insights into the common structural features and distinct conformational changes in the interaction patterns of sulfatinib with FGFR1 and CSF-1R, shedding light on the binding mechanism and kinase selectivity of sulfatinib. Guided by the structure-based drug design, we provided valuable perspectives for the rational design of dual- or multi-target FGFR inhibitors with enhanced CSF-1R selectivity and gatekeeper mutation sensitivity. These findings pave the way for future advancement in developing more effective therapies targeting FGFR and CSF-1R.

## Methods

### Expression and purification of the human FGFR kinase domain

FGFRs were prepared as previously described^6,34,44,45^. Briefly, FGFR1 (residues 458–765), FGFR2 (residues 458–768), FGFR3 (residues 450–758) as well as FGFR4 (residues 445–753), and their mutants, FGFR1^C584S^, FGFR1^V561M^, FGFR2^V564I^, FGFR2^V564F^ and FGFR3^V555M^, were cloned into a modified pET28a vector in frame with an N-terminal PreScission-cleavable 6×His tag and expressed in E. coli BL21 Rosetta cells. For crystallization, FGFR1^C584S^ was co-expressed with untagged YOPH to induce non-phosphorylated proteins. The harvested cell pellets were lysed in a buffer containing 20 mM Tris-HCl, pH 8.0, 500 mM NaCl, 20 mM imidazole and 0.5 mM TCEP, and FGFRs were purified over Ni-NTA resin followed by enzymatic digestion with PreScission for 6×His-tag cleavage and further purified by anion exchange chromatography (GE Healthcare). For crystallization, FGFR1^C584S^ was further purified by gel filtration chromatography (GE Healthcare). The proteins were concentrated to 5–16 mg/mL and stored at −80 °C.

### Expression and purification of the human CSF-1R kinase domain

Wild-type CSF-1R (residues 542–919, △696–741, C677T, C830S, C907T) and CSF-1R^T663I^ carrying an N-terminal 6×His tag was cloned into a pFastBac expression vector, and was expressed in SF9 cells using the Invitrogen Bac-to-Bac Baculovirus Expression System^41^. For crystallization, CSF-1R was co-expressed with N-terminal GST-tagged YOPH to induce non-phosphorylated proteins. The harvested cell pellets were lysed by sonication in lysis buffer consisting of 40 mM K-phosphate, pH 8.0, 200 mM NaCl, 0.5 mM PMSF, and protease inhibitor mixture (EDTA-free, Roche). After centrifugation, the clarified cell lysate was supplemented with 20 mM imidazole, and incubated with Ni-NTA beads (GE Healthcare). The Ni-NTA bead-bound CSF-1R protein was eluted using 25 mM HEPES, pH 7.0, 150 mM NaCl and 200 mM imidazole, and was subsequently supplemented with 10 mM DTT after elution. CSF-1R was further purified using a cation exchange column (GE Healthcare) followed by a Superdex200 gel filtration column (GE Healthcare) equilibrated in 20 mM HEPES, pH 7.0, 150 mM NaCl and 10 mM DTT.

### Crystallization and structure determination

The FGFR1/sulfatinib complex was obtained by micro-seeding as previously described^34^. FGFR1 was diluted 1:1 with reservoir solution of 18% (w/v) PEG 8000, 0.2 M LiSO_4_, and 0.1 M MES, pH 6.5, using the hanging drop vapor diffusion method. The apo FGFR1 crystals were allowed to grow for at least one week at 4 °C. For micro-seeding, FGFR1 was incubated with sulfatinib at a ratio of 1:2 at 4 °C overnight, and diluted 1:1 with the same reservoir solution as that of apo FGFR1 with the addition of apo FGFR1 microcrystals. The CSF-1R protein, at a concentration of ~10 mg/mL, was incubated with 1 mM sulfatinib before suspending over a reservoir solution of 0.1 M Tris, pH 7.5, 0.2 M MgCl_2_ and 20% (w/v) PEG 8000 at 4 °C. Both FGFR1/sulfatinib and CSF-1R/sulfatinib crystals were cryoprotected using their reservoir solution supplemented with 20% glycerol, followed by flash-freezing with liquid nitrogen.

X-ray diffraction data of CSF-1R/sulfatinib crystals were collected at the BL19U1 beamline of Shanghai Synchrotron Radiation Facility (SSRF), while FGFR1/sulfatinib-crystal datasets were collected in our lab with HKL3000 for data processing and scaling^46^. The structures were solved by molecular replacement using Phaser with the search model of CSF-1R/PLX-647OME (PDB 4HW7) for CSF-1R/sulfatinib and FGFR1/pemigatinib (PDB 7WCL) for FGFR1/sulfatinib^32,34,41^. Further, the models were refined with Phenix. refine and build with Coot^46^. The protein–ligand interactions were assessed by LigPlot+ and presented by PyMOL^47,48^. The structures have been deposited in the Protein Data Bank (PDB 8JOT for CSF-1R/sulfatinib and 8JMZ for FGFR1/sulfatinib).

### Kinase inhibition assay

The inhibition by sulfatinib was determined by the measurement of kinase activities following the ADP-Glo assay kit (Promega)^6,44,45^. Assays were performed in 384-well plates using sulfatinib (triple dilution method), proteins (0.025–0.2 μM), and poly (4:1 Glu, Tyr) peptides (Abcam, UK) (1 mg/mL) in assay buffer (40 mM Tris-HCl, pH 7.5, 20 mM NaCl, 20 mM MgCl_2_, 1 mM TCEP, 0.1 mg/mL BSA, and 4% DMSO). The reaction was initiated by the addition of ATP into the assay mixture, and then ADP-Glo was added to terminate the reaction and deplete the remaining ATP. Following the addition of detection reagent to convert the generated ADP to ATP, the luminescence was detected by a plate reader (Perkin Elmer). Samples were run in triplicate and the IC_50_ values were calculated by log[Inhibitor] versus kinase activity ratio in GraphPad Prism 8.0.

### Molecular modeling

Computational docking was conducted to evaluate the binding models of sulfatinib with CSF-1R mirroring our solved FGFR1-sulfatinib-like and CSF-1R-sulfatinib-like binding modes. Sulfatinib was redocked into CSF-1R (PDB 8JOT). Gasteiger charges and polar hydrogen were assigned by AutoDock Tools^49^. Molecular docking was performed with a flexible protein and a flexible ligand. A docking grid was built with the dimensions of 62*40*40 points in the x-, y-, and z-axis directions, which incorporated both FGFR1-bound and CSF-1R-bound sulfatinib conformations. Likewise, PRN1371 was docked into CSF-1R (PDB 3LCD)^50^. Additionally, the structural models of FGFR and CSF-1R gatekeeper mutations in complex with sulfatinib were prepared by protein mutagenesis based on our solved FGFR1 and CSF-1R structures.

### Reporting summary

Further information on research design is available in the Nature Portfolio Reporting Summary linked to this article.

### Supplementary information

Supplementary Information

Description of Additional Supplementary Files

Supplementary Data 1

Supplementary Data 2

Reporting Summary

## Data Availability

The coordinates and structure factors are deposited in the Protein Data Bank under the accession codes 8JMZ (FGFR1/sulfatinib complex) and 8JOT (CSF-1R/sulfatinib complex). The validation reports are available in Supplementary Data 1 and 2. All other relevant data supporting the key findings of this study are available within the article and its Supplementary Information files. A reporting summary is available as a Supplementary Information file.
