# Supplementary material for: Structural basis and selectivity of sulfatinib binding to FGFR and CSF-1R
Source: Commun Chem. 2024 Jan 3;7:3. doi: 10.1038/s42004-023-01084-0 (PMC10764862; doi:10.1038/s42004-023-01084-0)
Supplement: Supplementary file 1 — Supplementary Information [file 42004_2023_1084_MOESM1_ESM.pdf]

Supplementary Tables and Figures

Supplementary Tables

Supplementary Table 1. Statistical parameters for data collection and structure refinement

	FGFR1/Sulfatinib[a]	CSF-1R/Sulfatinib[b]
**PDB code**	8JMZ	8JOT
**Data collection**		
Space group	C 1 2 1	P 43 21 2
Cell dimensions		
a, b, c (Å)	211.46, 49.87, 66.69	64.31, 64.31, 184.69
α, β, γ (°)	90.00, 107.57, 90.00	90.00, 90.00, 90.00
Resolution (Å)	37.24 - 1.99 (2.06 - 1.99)	36.58 - 1.69 (1.75 - 1.69)
R_{merge}	0.07 (0.32)	0.21 (0.75)
$I / \sigma I$	19.43 (4.08)	9.20 (1.70)
Completeness (%)	100 (96)	99.61 (96.56)
Redundancy	6.8 (5.2)	19.9 (6.7)
**Refinement**		
Resolution (Å)	37.24 - 1.99	36.58 - 1.69
No. reflections	45818 (4353)	44269 (4188)
R_{work} / R_{free}	0.16/0.20	0.18/0.21
No. atoms		
Protein	598	306
Ligand/ion	125	40
Water	455	312
B-factors		
Protein	30.18	28.65
Ligand/ion	44.70	37.20
Water	36.67	40.87
R.m.s. deviations		
Bond lengths (Å)	0.030	0.007
Bond angles (°)	1.38	0.82

[a]The X-ray diffraction data of FGFR1/sulfatinib were collected in our lab with source of MicroMax-007HF. [b]CSF-1R/sulfatinib data were collected at the BL19U1 beamline of Shanghai Synchrotron Radiation Facility (SSRF).

Supplementary Figures

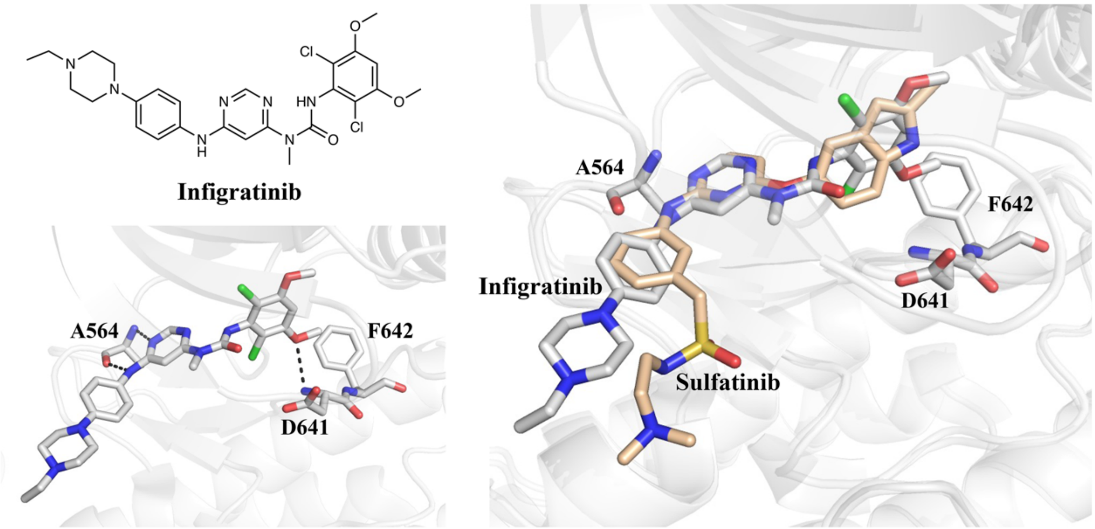

Supplementary Figure 1. Structural comparison of FGFR1 in complex with sulfatinib and infigratinib. (A) Chemical structure of infigratinib. (B) Hydrogen-bond interactions between infigratinib and FGFR1 (PDB 3TT0). (C) Superposition of sulfatinib (wheat) and infigratinib (grey) upon FGFR1 binding.

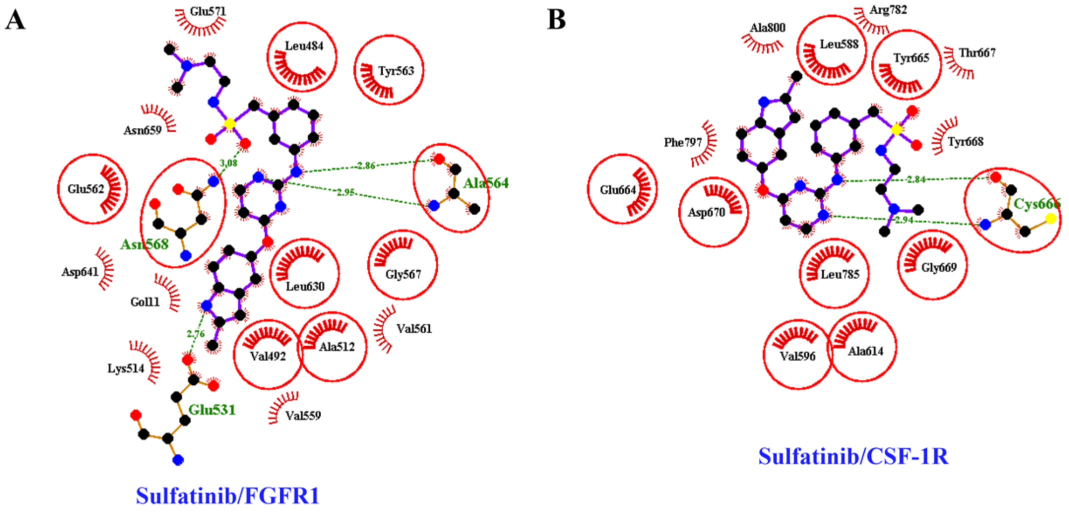

Supplementary Figure 2. Ligand interaction diagrams (LIDs) demonstrate the interaction patterns of FGFR1/sulfatinib (A) and CSF-1R/sulatinib (B). Hydrogen bonds (distance ≤ 3.35 Å) are designated with a green dashed line, and van der Waals interactions (distance ≤ 3.9 Å) are shown by a red half "sun" arrangement. The aligned residues are circled in red. Graphics were drawn by LigPlot+.

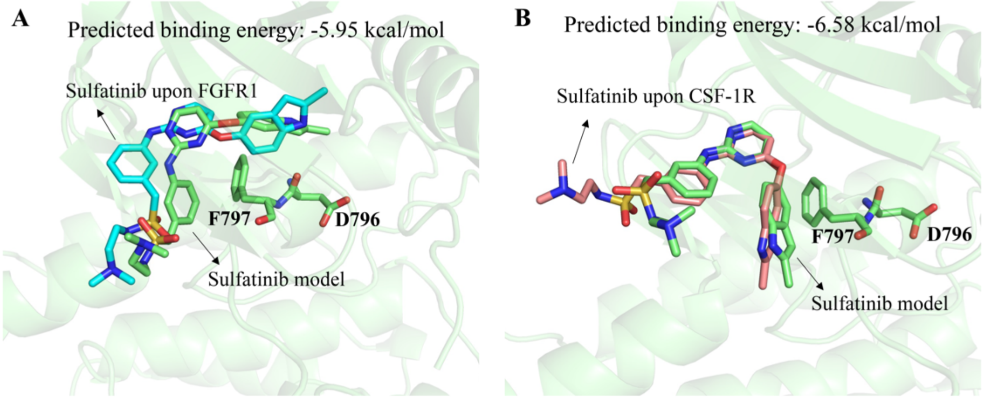

FGFR1/sulfatinib-like binding model   CSF-1R/sulfatinib-like binding model

Supplementary Figure 3. Sulfatinib tends to flip out of the hydrophobic pocket when binding to CSF-1R. (A) The predicted binding model of sulfatinib (green) occupies the hydrophobic pocket upon CSF-1R binding with higher predicted binding energy, and is aligned with our solved structure of FGFR1-bound sulfatinib (cyan). (B) Predicted binding model of sulfatinib/CSF-1R (green) resembles our solved structure of CSF-1R-bound sulfatinib (salmon), which presents a relatively lower predicted binding energy.

**FGFR1**	[556]	PLYVIVEYASKGN
**FGFR2**	[559]	PLYVIVEYASKGN
**FGFR3**	[550]	PLYVLVEYAAKGN
**CSF-1R**	[658]	PVLVITEYCCYGD
**c-KIT**	[665]	PTLVITEYCCYGD
**PDGFRα**	[669]	PIYIITEYCFYGD
**BCR-ABL**	[310]	PFYIVTEYMPYGN

Supplementary Figure 4. Sequence alignment of the gatekeeper residues among kinase proteins. The position of the gatekeeper residue is colored red.

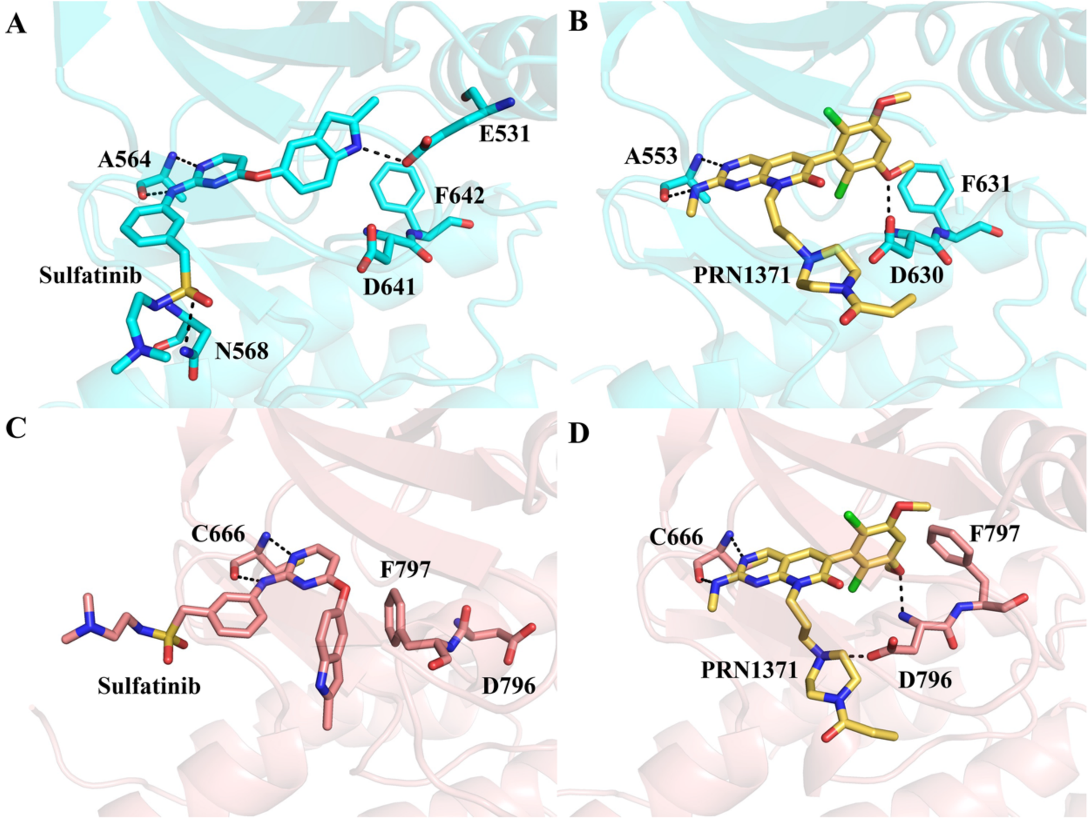

Supplementary Figure 5. Structural comparisons of sulfatinib and PRN1371 binding to FGFR and CSF-1R. (A) Crystal structure of sulfatinib in complex with FGFR1 (PDB 8JMZ). (B) Crystal structure of PRN1371 in complex with FGFR4 (PDB 7F3M). (C) Crystal structure of sulfatinib in complex with CSF-1R (PDB 8JOT). (D) Predicted binding mode of PRN1371 in complex with CSF-1R based on crystal of CSF-1R/compound **3a** (PDB 3LCD). FGFR is colored cyan, while CSF-1R is colored salmon.
