# Supplementary material for: Structural basis and selectivity of sulfatinib binding to FGFR and CSF-1R
Source: Commun Chem. 2024 Jan 3;7:3. doi: 10.1038/s42004-023-01084-0 (PMC10764862; doi:10.1038/s42004-023-01084-0)
Supplement: Supplementary file 2 — Description of Additional Supplementary Files [file 42004_2023_1084_MOESM2_ESM.docx]

Description of Additional Supplementary Files

**File name: Supplementary Data 1**

**Description: Validation report of the crystal structure of FGFR1/Sulfatinib complex (PDB 8JMZ)**

**File name: Supplementary Data 2**

**Description: Validation report of the crystal structure of CSF-1R/Sulfatinib complex (PDB 8JOT)**
