# Supplementary material for: Structural basis and selectivity of sulfatinib binding to FGFR and CSF-1R
Source: Commun Chem. 2024 Jan 3;7:3. doi: 10.1038/s42004-023-01084-0 (PMC10764862; doi:10.1038/s42004-023-01084-0)
Supplement: Supplementary file 3 — Supplementary Data 1 [file 42004_2023_1084_MOESM3_ESM.zip › supplementary data 1/sulfatinib_FGFR1_val-report-full-annotate_P1.pdf]

Full wwPDB X-ray Structure Validation Report (i)

Jun 8, 2023 – 03:29 PM JST

PDB ID	:	8JMZ
Title	:	FGFR1 kinase domain with sulfatinib
Deposited on	:	2023-06-05
Resolution	:	1.99 Å(reported)

This wwPDB validation report is for manuscript review

This is a Full wwPDB X-ray Structure Validation Report.

This report is produced by the wwPDB biocuration pipeline after annotation of the structure.

We welcome your comments at *validation@mail.wwpdb.org*
A user guide is available at
https://www.wwpdb.org/validation/2017/XrayValidationReportHelp
with specific help available everywhere you see the (i) symbol.

The types of validation reports are described at
http://www.wwpdb.org/validation/2017/FAQs#types.

For Manuscript Review

The following versions of software and data (see references (i)) were used in the production of this report:

MolProbity	:	4.02b-467
Mogul	:	1.8.5 (274361), CSD as541be (2020)
Xtriage (Phenix)	:	1.13
EDS	:	2.33
buster-report	:	1.1.7 (2018)
Percentile statistics	:	20191225.v01 (using entries in the PDB archive December 25th 2019)
Refmac	:	5.8.0158
CCP4	:	7.0.044 (Gargrove)
Ideal geometry (proteins)	:	Engh & Huber (2001)
Ideal geometry (DNA, RNA)	:	Parkinson et al. (1996)

1 Overall quality at a glance (i)

The following experimental techniques were used to determine the structure:
X-RAY DIFFRACTION

The reported resolution of this entry is 1.99 Å.

Percentile scores (ranging between 0-100) for global validation metrics of the entry are shown in the following graphic. The table shows the number of entries on which the scores are based.

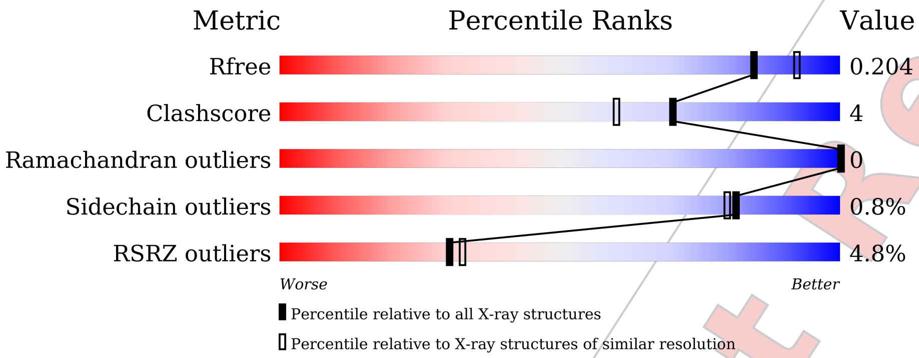

Metric	Whole archive (#Entries)	Similar resolution (#Entries, resolution range(Å))
R_{free}	130704	11647 (2.00-1.96)
Clashscore	141614	1014 (1.98-1.98)
Ramachandran outliers	138981	1006 (1.98-1.98)
Sidechain outliers	138945	1006 (1.98-1.98)
RSRZ outliers	127900	11410 (2.00-1.96)

The table below summarises the geometric issues observed across the polymeric chains and their fit to the electron density. The red, orange, yellow and green segments of the lower bar indicate the fraction of residues that contain outliers for >=3, 2, 1 and 0 types of geometric quality criteria respectively. A grey segment represents the fraction of residues that are not modelled. The numeric value for each fraction is indicated below the corresponding segment, with a dot representing fractions <=5% The upper red bar (where present) indicates the fraction of residues that have poor fit to the electron density. The numeric value is given above the bar.

Mol	Chain	Length	Quality of chain
1	A	310	3% / 94% • •
1	B	310	6% / 87% 7% 6%

For Manuscript Review

2 Entry composition (i)

There are 5 unique types of molecules in this entry. The entry contains 5278 atoms, of which 0 are hydrogens and 0 are deuteriums.

In the tables below, the ZeroOcc column contains the number of atoms modelled with zero occupancy, the AltConf column contains the number of residues with at least one atom in alternate conformation and the Trace column contains the number of residues modelled with at most 2 atoms.

- Molecule 1 is a protein called Fibroblast growth factor receptor 1.

Mol	Chain	Residues	Atoms					ZeroOcc	AltConf	Trace
			Total	C	N	O	S			
1	A	306	2418	1535	411	454	18	0	0	0
1	B	292	2290	1456	395	421	18	0	1	0

There are 6 discrepancies between the modelled and reference sequences:

Chain	Residue	Modelled	Actual	Comment	Reference
A	456	GLY	-	expression tag	UNP P11362
A	457	PRO	-	expression tag	UNP P11362
A	584	SER	CYS	engineered mutation	UNP P11362
B	456	GLY	-	expression tag	UNP P11362
B	457	PRO	-	expression tag	UNP P11362
B	584	SER	CYS	engineered mutation	UNP P11362

- Molecule 2 is N-(2-(dimethylamino)ethyl)-1-(3-((4-((2-methyl-1H-indol-5-yl)oxy)pyrimidin-2-yl)amino)phenyl)methanesulfonamide (three-letter code: UKI) (formula: $C_{24}H_{28}N_6O_3S$) (labeled as "Ligand of Interest" by depositor).

For Manuscript Review

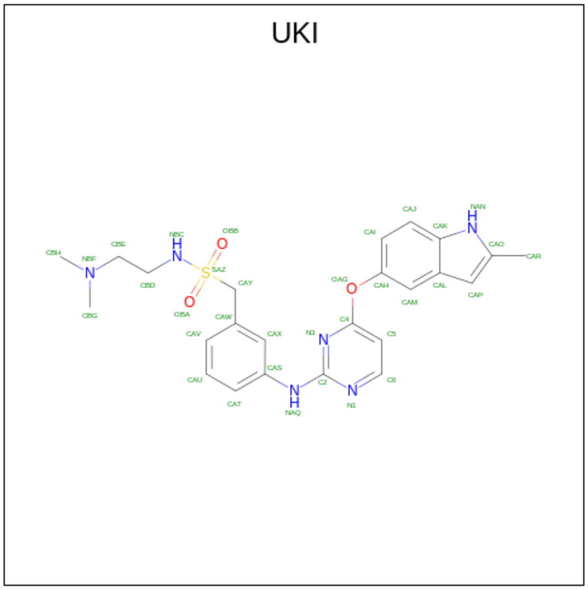

Mol	Chain	Residues	Atoms					ZeroOcc	AltConf
			Total	C	N	O	S		
2	A	1	34	24	6	3	1	0	0
2	B	1	34	24	6	3	1	0	0

- Molecule 3 is SULFATE ION (three-letter code: SO4) (formula: O_4S).

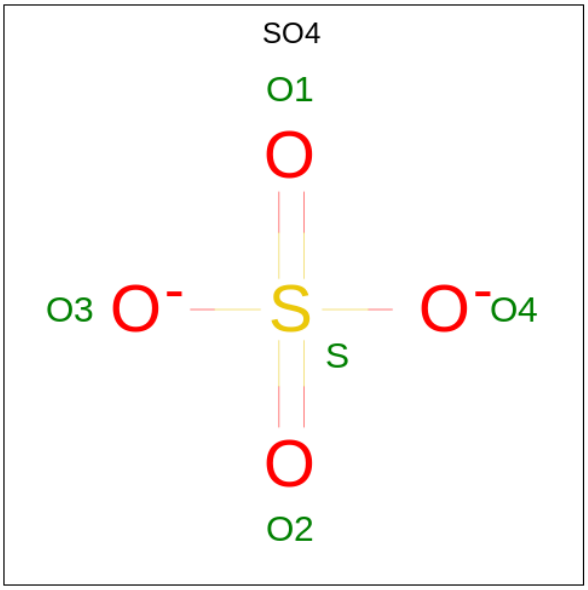

Mol	Chain	Residues	Atoms			ZeroOcc	AltConf
			Total	O	S		
3	A	1	5	4	1	0	0
3	A	1	5	4	1	0	0

Continued on next page...

For Manuscript Review

Continued from previous page...

Mol	Chain	Residues	Atoms	ZeroOcc	AltConf
3	A	1	Total O S<br>5 4 1	0	0
3	A	1	Total O S<br>5 4 1	0	0
3	A	1	Total O S<br>5 4 1	0	0
3	A	1	Total O S<br>5 4 1	0	0
3	B	1	Total O S<br>5 4 1	0	0
3	B	1	Total O S<br>5 4 1	0	0
3	B	1	Total O S<br>5 4 1	0	0

- Molecule 4 is GLYCEROL (three-letter code: GOL) (formula: $C_3H_8O_3$).

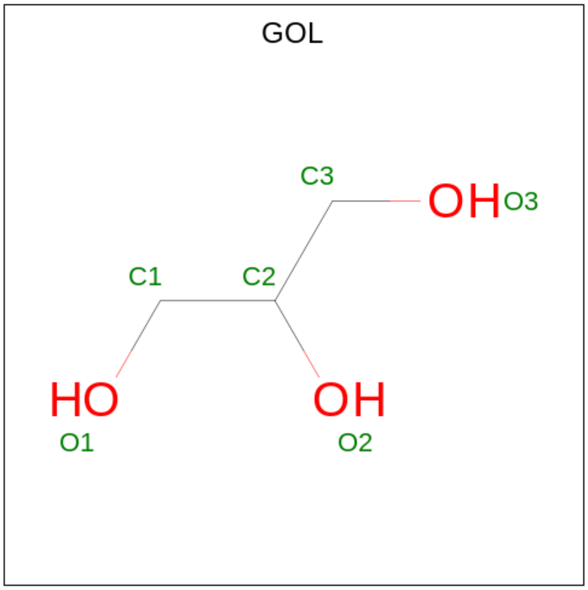

Mol	Chain	Residues	Atoms	ZeroOcc	AltConf
4	A	1	Total C O<br>6 3 3	0	0
4	B	1	Total C O<br>6 3 3	0	0

- Molecule 5 is water.

For Manuscript Review

Mol	Chain	Residues	Atoms	ZeroOcc	AltConf
5	A	235	Total O<br>235 235	0	0
5	B	210	Total O<br>210 210	0	0

For Manuscript Review

3 Residue-property plots (i)

These plots are drawn for all protein, RNA, DNA and oligosaccharide chains in the entry. The first graphic for a chain summarises the proportions of the various outlier classes displayed in the second graphic. The second graphic shows the sequence view annotated by issues in geometry and electron density. Residues are color-coded according to the number of geometric quality criteria for which they contain at least one outlier: green = 0, yellow = 1, orange = 2 and red = 3 or more. A red dot above a residue indicates a poor fit to the electron density (RSRZ > 2). Stretches of 2 or more consecutive residues without any outlier are shown as a green connector. Residues present in the sample, but not in the model, are shown in grey.

- Molecule 1: Fibroblast growth factor receptor 1

Chain A:

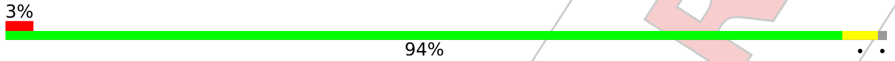

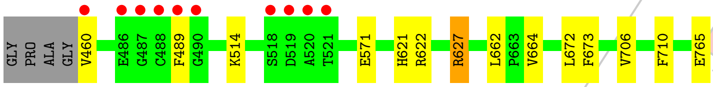

- Molecule 1: Fibroblast growth factor receptor 1

Chain B:

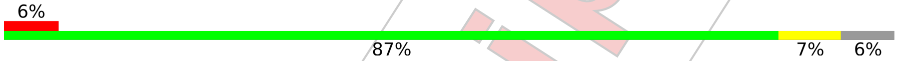

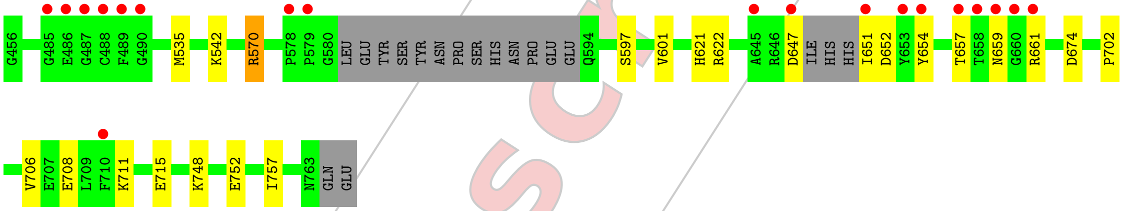

For Manuscript Review

4 Data and refinement statistics (i)

Property	Value	Source
Space group	C 1 2 1	Depositor
Cell constants<br>a, b, c, α, β, γ	211.46Å 49.87Å 66.69Å<br>90.00° 107.57° 90.00°	Depositor
Resolution (Å)	40.05 – 1.99<br>40.05 – 1.99	Depositor<br>EDS
% Data completeness<br>(in resolution range)	99.6 (40.05-1.99)<br>99.6 (40.05-1.99)	Depositor<br>EDS
R_{merge}	0.07	Depositor
R_{sym}	(Not available)	Depositor
$< I/\sigma(I) >$ [1]	5.60 (at 1.98Å)	Xtriage
Refinement program	PHENIX 1.9_1692	Depositor
R, R_{free}	0.163 , 0.205<br>0.165 , 0.204	Depositor<br>DCC
R_{free} test set	2356 reflections (5.14%)	wwPDB-VP
Wilson B-factor (Å^2)	26.4	Xtriage
Anisotropy	0.301	Xtriage
Bulk solvent k_{sol}(e/Å^3), B_{sol}(Å^2)	0.36 , 47.2	EDS
L-test for twinning[2]	$< \|L\| > = 0.50$, $< L^2 > = 0.33$	Xtriage
Estimated twinning fraction	0.014 for -h-2*l,-k,l	Xtriage
F_o,F_c correlation	0.96	EDS
Total number of atoms	5278	wwPDB-VP
Average B, all atoms (Å^2)	31.0	wwPDB-VP

Xtriage's analysis on translational NCS is as follows: *The largest off-origin peak in the Patterson function is 4.74% of the height of the origin peak. No significant pseudotranslation is detected.*

[1] Intensities estimated from amplitudes.
[2] Theoretical values of $< |L| >$, $< L^2 >$ for acentric reflections are 0.5, 0.333 respectively for untwinned datasets, and 0.375, 0.2 for perfectly twinned datasets.

For Manuscript Review

5 Model quality (i)

5.1 Standard geometry (i)

Bond lengths and bond angles in the following residue types are not validated in this section: SO4, GOL, UKI

The Z score for a bond length (or angle) is the number of standard deviations the observed value is removed from the expected value. A bond length (or angle) with $|Z| > 5$ is considered an outlier worth inspection. RMSZ is the root-mean-square of all Z scores of the bond lengths (or angles).

Mol	Chain	Bond lengths		Bond angles	
		RMSZ	#|Z| >5	RMSZ	#|Z| >5
1	A	0.62	0/2473	0.64	0/3354
1	B	0.55	0/2341	0.67	1/3168 (0.0%)
All	All	0.59	0/4814	0.65	1/6522 (0.0%)

Chiral center outliers are detected by calculating the chiral volume of a chiral center and verifying if the center is modelled as a planar moiety or with the opposite hand.A planarity outlier is detected by checking planarity of atoms in a peptide group, atoms in a mainchain group or atoms of a sidechain that are expected to be planar.

Mol	Chain	#Chirality outliers	#Planarity outliers
1	A	0	1
1	B	0	1
All	All	0	2

There are no bond length outliers.

All (1) bond angle outliers are listed below:

Mol	Chain	Res	Type	Atoms	Z	Observed(°)	Ideal(°)
1	B	659	ASN	CB-CA-C	6.01	122.43	110.40

There are no chirality outliers.

All (2) planarity outliers are listed below:

Mol	Chain	Res	Type	Group
1	A	627	ARG	Sidechain
1	B	570	ARG	Sidechain

For Manuscript Review

5.2 Too-close contacts (i)

In the following table, the Non-H and H(model) columns list the number of non-hydrogen atoms and hydrogen atoms in the chain respectively. The H(added) column lists the number of hydrogen atoms added and optimized by MolProbity. The Clashes column lists the number of clashes within the asymmetric unit, whereas Symm-Clashes lists symmetry-related clashes.

Mol	Chain	Non-H	H(model)	H(added)	Clashes	Symm-Clashes
1	A	2418	0	2375	8	0
1	B	2290	0	2276	23	0
2	A	34	0	0	0	0
2	B	34	0	0	0	0
3	A	30	0	0	0	0
3	B	15	0	0	0	0
4	A	6	0	8	1	0
4	B	6	0	8	2	0
5	A	235	0	0	2	0
5	B	210	0	0	7	0
All	All	5278	0	4667	34	0

The all-atom clashscore is defined as the number of clashes found per 1000 atoms (including hydrogen atoms). The all-atom clashscore for this structure is 4.

All (34) close contacts within the same asymmetric unit are listed below, sorted by their clash magnitude.

Atom-1	Atom-2	Interatomic distance (Å)	Clash overlap (Å)
1:B:570:ARG:HD3	1:B:661:ARG:NH2	1.86	0.89
1:A:664:VAL:HG23	1:A:672:LEU:HD11	1.66	0.78
1:B:570:ARG:NH1	1:B:661:ARG:CZ	2.56	0.68
4:A:808:GOL:H32	5:A:901:HOH:O	2.01	0.59
1:B:535:MET:HE3	5:B:1063:HOH:O	2.03	0.59
1:B:711:LYS:O	1:B:715:GLU:HG2	2.04	0.58
1:B:570:ARG:NH1	1:B:661:ARG:NE	2.52	0.58
1:A:460:VAL:N	5:A:906:HOH:O	2.38	0.56
1:B:570:ARG:CZ	1:B:661:ARG:NE	2.69	0.56
1:B:570:ARG:CD	1:B:661:ARG:NH2	2.65	0.55
4:B:805:GOL:H11	5:B:908:HOH:O	2.11	0.51
1:B:597:SER:O	1:B:601:VAL:HG23	2.11	0.51
1:B:570:ARG:HH11	1:B:661:ARG:CZ	2.21	0.51
1:A:571:GLU:OE2	1:A:627:ARG:NH2	2.36	0.50
1:A:664:VAL:CG2	1:A:672:LEU:HD11	2.39	0.49
1:B:708:GLU:OE2	5:B:901:HOH:O	2.20	0.49
4:B:805:GOL:C1	5:B:908:HOH:O	2.60	0.48

Continued on next page...

For Manuscript Review

Continued from previous page...

Atom-1	Atom-2	Interatomic distance (Å)	Clash overlap (Å)
1:A:662:LEU:HD13	1:A:706:VAL:HG21	1.95	0.48
1:B:654:TYR:HD2	1:B:706:VAL:HG11	1.78	0.47
1:B:542:LYS:HE3	5:B:1026:HOH:O	2.13	0.47
1:B:570:ARG:HD3	1:B:661:ARG:HH22	1.75	0.47
1:B:654:TYR:CD2	1:B:706:VAL:HG11	2.50	0.47
1:B:621:HIS:O	1:B:622:ARG:HB2	2.17	0.44
1:B:674:ASP:OD2	5:B:902:HOH:O	2.21	0.44
1:A:673:PHE:HB2	1:A:710:PHE:CE1	2.52	0.44
1:B:570:ARG:HH11	1:B:661:ARG:NH1	2.17	0.43
1:A:621:HIS:O	1:A:622:ARG:HB2	2.19	0.42
1:B:570:ARG:CZ	1:B:661:ARG:CZ	2.97	0.42
1:A:489:PHE:CE1	1:A:514:LYS:HE3	2.55	0.41
1:B:570:ARG:NH1	1:B:661:ARG:CD	2.83	0.41
1:B:601:VAL:HG11	1:B:757:ILE:HG22	2.02	0.41
1:B:748:LYS:NZ	1:B:752:GLU:OE2	2.52	0.40
1:B:702:PRO:HD2	5:B:914:HOH:O	2.21	0.40
1:B:657:THR:N	1:B:661:ARG:O	2.51	0.40

There are no symmetry-related clashes.

5.3 Torsion angles (i)

5.3.1 Protein backbone (i)

In the following table, the Percentiles column shows the percent Ramachandran outliers of the chain as a percentile score with respect to all X-ray entries followed by that with respect to entries of similar resolution.

The Analysed column shows the number of residues for which the backbone conformation was analysed, and the total number of residues.

Mol	Chain	Analysed	Favoured	Allowed	Outliers	Percentiles	
1	A	304/310 (98%)	299 (98%)	5 (2%)	0	100	100
1	B	287/310 (93%)	284 (99%)	3 (1%)	0	100	100
All	All	591/620 (95%)	583 (99%)	8 (1%)	0	100	100

There are no Ramachandran outliers to report.

For Manuscript Review

5.3.2 Protein sidechains (i)

In the following table, the Percentiles column shows the percent sidechain outliers of the chain as a percentile score with respect to all X-ray entries followed by that with respect to entries of similar resolution.

The Analysed column shows the number of residues for which the sidechain conformation was analysed, and the total number of residues.

Mol	Chain	Analysed	Rotameric	Outliers	Percentiles	
1	A	263/271 (97%)	262 (100%)	1 (0%)	91	90
1	B	246/271 (91%)	243 (99%)	3 (1%)	71	67
All	All	509/542 (94%)	505 (99%)	4 (1%)	81	80

All (4) residues with a non-rotameric sidechain are listed below:

Mol	Chain	Res	Type
1	A	765	GLU
1	B	647	ASP
1	B	651	ILE
1	B	652	ASP

Sometimes sidechains can be flipped to improve hydrogen bonding and reduce clashes. There are no such sidechains identified.

5.3.3 RNA (i)

There are no RNA molecules in this entry.

5.4 Non-standard residues in protein, DNA, RNA chains (i)

There are no non-standard protein/DNA/RNA residues in this entry.

5.5 Carbohydrates (i)

There are no monosaccharides in this entry.

5.6 Ligand geometry (i)

13 ligands are modelled in this entry.

For Manuscript Review

In the following table, the Counts columns list the number of bonds (or angles) for which Mogul statistics could be retrieved, the number of bonds (or angles) that are observed in the model and the number of bonds (or angles) that are defined in the Chemical Component Dictionary. The Link column lists molecule types, if any, to which the group is linked. The Z score for a bond length (or angle) is the number of standard deviations the observed value is removed from the expected value. A bond length (or angle) with $|Z| > 2$ is considered an outlier worth inspection. RMSZ is the root-mean-square of all Z scores of the bond lengths (or angles).

Mol	Type	Chain	Res	Link	Bond lengths			Bond angles		
					Counts	RMSZ	$\#\|Z\| > 2$	Counts	RMSZ	$\#\|Z\| > 2$
3	SO4	A	805	-	4,4,4	0.14	0	6,6,6	0.11	0
4	GOL	B	805	-	5,5,5	0.42	0	5,5,5	1.02	0
2	UKI	B	801	-	34,37,37	2.63	9 (26%)	45,52,52	2.37	8 (17%)
3	SO4	A	806	-	4,4,4	0.32	0	6,6,6	0.73	0
3	SO4	B	803	-	4,4,4	0.54	0	6,6,6	0.59	0
3	SO4	A	802	-	4,4,4	0.15	0	6,6,6	0.27	0
3	SO4	A	807	-	4,4,4	0.25	0	6,6,6	0.34	0
3	SO4	B	802	-	4,4,4	0.14	0	6,6,6	0.21	0
3	SO4	A	804	-	4,4,4	0.14	0	6,6,6	0.24	0
3	SO4	A	803	-	4,4,4	0.16	0	6,6,6	0.19	0
4	GOL	A	808	-	5,5,5	0.14	0	5,5,5	0.37	0
3	SO4	B	804	-	4,4,4	0.15	0	6,6,6	0.11	0
2	UKI	A	801	-	34,37,37	2.52	9 (26%)	45,52,52	2.64	11 (24%)

In the following table, the Chirals column lists the number of chiral outliers, the number of chiral centers analysed, the number of these observed in the model and the number defined in the Chemical Component Dictionary. Similar counts are reported in the Torsion and Rings columns. '-' means no outliers of that kind were identified.

Mol	Type	Chain	Res	Link	Chirals	Torsions	Rings
4	GOL	B	805	-	-	2/4/4/4	-
2	UKI	B	801	-	-	3/20/20/20	0/4/4/4
2	UKI	A	801	-	-	5/20/20/20	0/4/4/4
4	GOL	A	808	-	-	4/4/4/4	-

All (18) bond length outliers are listed below:

Mol	Chain	Res	Type	Atoms	Z	Observed(Å)	Ideal(Å)
2	B	801	UKI	OBA-SAZ	7.58	1.54	1.43
2	B	801	UKI	OBB-SAZ	7.38	1.54	1.43
2	A	801	UKI	OBA-SAZ	7.27	1.53	1.43
2	A	801	UKI	OBB-SAZ	6.42	1.52	1.43
2	B	801	UKI	CAY-CAW	-5.98	1.40	1.50

Continued on next page...

For Manuscript Review

Continued from previous page...

Mol	Chain	Res	Type	Atoms	Z	Observed(Å)	Ideal(Å)
2	A	801	UKI	CAY-CAW	-5.82	1.41	1.50
2	B	801	UKI	CAJ-CAK	-4.49	1.34	1.41
2	A	801	UKI	CAJ-CAK	-4.40	1.34	1.41
2	B	801	UKI	CAP-CAO	-4.21	1.33	1.39
2	A	801	UKI	SAZ-NBC	4.20	1.72	1.61
2	B	801	UKI	SAZ-NBC	4.00	1.71	1.61
2	A	801	UKI	CAP-CAO	-3.91	1.34	1.39
2	A	801	UKI	CAM-CAL	-2.88	1.35	1.42
2	B	801	UKI	CAM-CAL	-2.78	1.35	1.42
2	B	801	UKI	CAM-CAH	2.55	1.41	1.37
2	B	801	UKI	C6-N1	2.32	1.39	1.34
2	A	801	UKI	CAJ-CAI	2.14	1.41	1.36
2	A	801	UKI	C6-N1	2.06	1.38	1.34

All (19) bond angle outliers are listed below:

Mol	Chain	Res	Type	Atoms	Z	Observed(^{o})	Ideal(^{o})
2	A	801	UKI	N1-C2-N3	-8.72	118.29	126.55
2	A	801	UKI	C6-N1-C2	8.17	122.70	115.45
2	B	801	UKI	N1-C2-N3	-7.69	119.27	126.55
2	B	801	UKI	C6-N1-C2	6.38	121.10	115.45
2	B	801	UKI	OBB-SAZ-OBA	-6.27	110.26	119.35
2	A	801	UKI	C2-N3-C4	6.07	122.13	114.99
2	B	801	UKI	C2-N3-C4	5.82	121.83	114.99
2	A	801	UKI	OBB-SAZ-OBA	-5.33	111.63	119.35
2	A	801	UKI	C5-C6-N1	-4.60	118.25	123.96
2	B	801	UKI	C5-C6-N1	-4.29	118.64	123.96
2	B	801	UKI	CBD-NBC-SAZ	-4.00	110.49	122.55
2	A	801	UKI	CBD-NBC-SAZ	-3.12	113.14	122.55
2	B	801	UKI	OAG-C4-C5	3.02	121.41	115.35
2	A	801	UKI	OAG-C4-C5	2.94	121.25	115.35
2	A	801	UKI	CAP-CAL-CAK	2.64	108.58	106.27
2	A	801	UKI	CAI-CAH-CAM	-2.55	117.38	120.81
2	A	801	UKI	NAQ-C2-N3	2.46	125.27	116.92
2	B	801	UKI	NAQ-C2-N3	2.39	125.02	116.92
2	A	801	UKI	CBE-CBD-NBC	2.35	114.67	110.20

There are no chirality outliers.

All (14) torsion outliers are listed below:

Mol	Chain	Res	Type	Atoms
2	A	801	UKI	NBC-CBD-CBE-NBF

Continued on next page...

For Manuscript Review

Continued from previous page...

Mol	Chain	Res	Type	Atoms
2	A	801	UKI	CBD-NBC-SAZ-OBA
2	A	801	UKI	CBD-NBC-SAZ-CAY
2	B	801	UKI	NBC-CBD-CBE-NBF
2	B	801	UKI	CBD-NBC-SAZ-OBA
4	B	805	GOL	O1-C1-C2-C3
4	B	805	GOL	O1-C1-C2-O2
4	A	808	GOL	O1-C1-C2-C3
4	A	808	GOL	C1-C2-C3-O3
4	A	808	GOL	O1-C1-C2-O2
4	A	808	GOL	O2-C2-C3-O3
2	B	801	UKI	CBD-NBC-SAZ-CAY
2	A	801	UKI	CBD-CBE-NBF-CBH
2	A	801	UKI	CBD-CBE-NBF-CBG

There are no ring outliers.

2 monomers are involved in 3 short contacts:

Mol	Chain	Res	Type	Clashes	Symm-Clashes
4	B	805	GOL	2	0
4	A	808	GOL	1	0

The following is a two-dimensional graphical depiction of Mogul quality analysis of bond lengths, bond angles, torsion angles, and ring geometry for all instances of the Ligand of Interest. In addition, ligands with molecular weight > 250 and outliers as shown on the validation Tables will also be included. For torsion angles, if less then 5% of the Mogul distribution of torsion angles is within 10 degrees of the torsion angle in question, then that torsion angle is considered an outlier. Any bond that is central to one or more torsion angles identified as an outlier by Mogul will be highlighted in the graph. For rings, the root-mean-square deviation (RMSD) between the ring in question and similar rings identified by Mogul is calculated over all ring torsion angles. If the average RMSD is greater than 60 degrees and the minimal RMSD between the ring in question and any Mogul-identified rings is also greater than 60 degrees, then that ring is considered an outlier. The outliers are highlighted in purple. The color gray indicates Mogul did not find sufficient equivalents in the CSD to analyse the geometry.

For Manuscript Review

Ligand UKI B 801

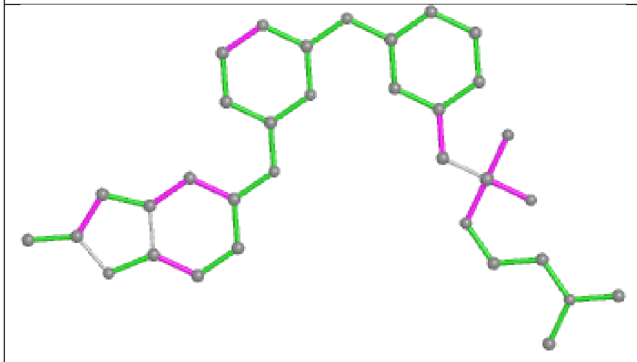

Bond lengths

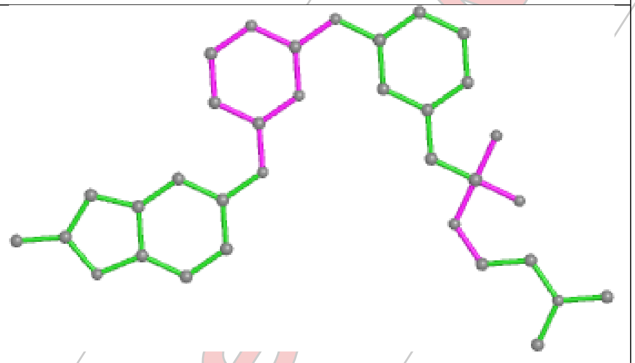

Bond angles

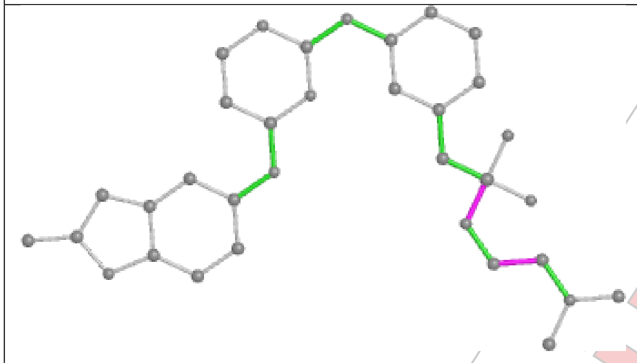

Torsions

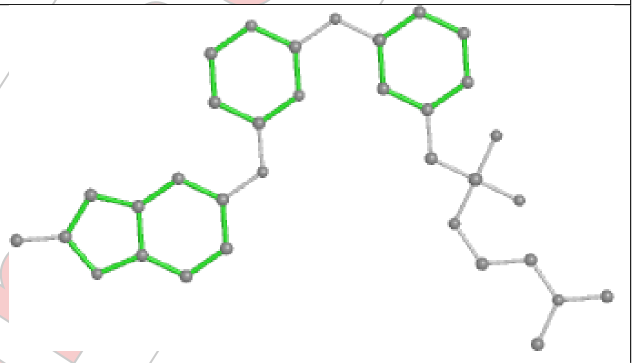

Rings

Ligand UKI A 801

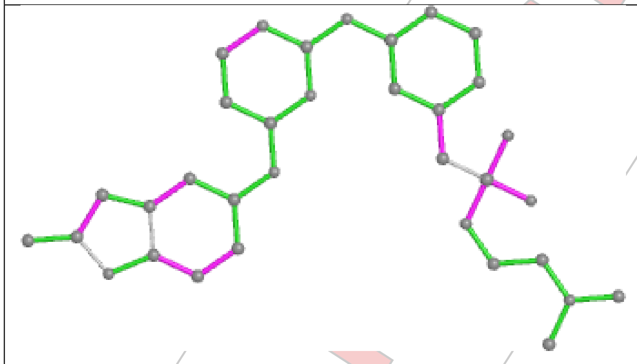

Bond lengths

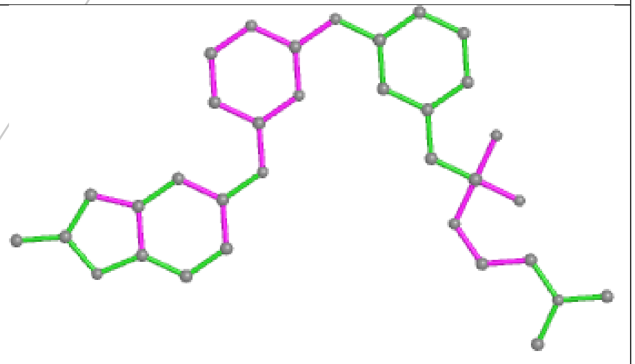

Bond angles

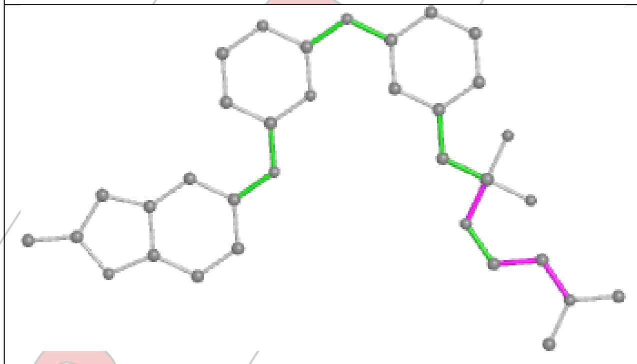

Torsions

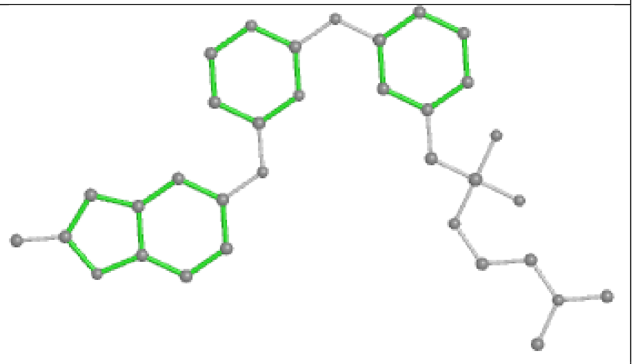

Rings

5.7 Other polymers (i)

There are no such residues in this entry.

5.8 Polymer linkage issues (i)

There are no chain breaks in this entry.

For Manuscript Review

6 Fit of model and data (i)

6.1 Protein, DNA and RNA chains (i)

In the following table, the column labelled '#RSRZ> 2' contains the number (and percentage) of RSRZ outliers, followed by percent RSRZ outliers for the chain as percentile scores relative to all X-ray entries and entries of similar resolution. The OWAB column contains the minimum, median, 95th percentile and maximum values of the occupancy-weighted average B-factor per residue. The column labelled 'Q< 0.9' lists the number of (and percentage) of residues with an average occupancy less than 0.9.

Mol	Chain	Analysed	<RSRZ>	#RSRZ>2			OWAB(Å^2)	Q<0.9
1	A	306/310 (98%)	-0.09	10 (3%)	46	49	17, 25, 51, 81	0
1	B	292/310 (94%)	-0.11	19 (6%)	18	20	18, 28, 64, 81	0
All	All	598/620 (96%)	-0.10	29 (4%)	30	32	17, 27, 56, 81	0

All (29) RSRZ outliers are listed below:

Mol	Chain	Res	Type	RSRZ
1	B	659	ASN	6.9
1	A	488	CYS	6.6
1	B	653	TYR	6.4
1	B	488	CYS	5.5
1	A	489	PHE	4.5
1	B	654	TYR	4.2
1	A	490	GLY	4.0
1	B	658	THR	4.0
1	A	487	GLY	3.8
1	B	485	GLY	3.6
1	A	519	ASP	3.5
1	B	486	GLU	3.5
1	B	647	ASP	3.3
1	B	660	GLY	3.2
1	A	486	GLU	3.2
1	B	490	GLY	3.1
1	A	460	VAL	3.1
1	B	489	PHE	3.1
1	A	520	ALA	2.9
1	B	579	PRO	2.7
1	B	657	THR	2.7
1	B	710	PHE	2.5
1	A	521	THR	2.4
1	A	518	SER	2.4

Continued on next page...

For Manuscript Review

Continued from previous page...

Mol	Chain	Res	Type	RSRZ
1	B	578	PRO	2.3
1	B	661	ARG	2.3
1	B	651	ILE	2.2
1	B	487	GLY	2.1
1	B	645	ALA	2.0

6.2 Non-standard residues in protein, DNA, RNA chains (i)

There are no non-standard protein/DNA/RNA residues in this entry.

6.3 Carbohydrates (i)

There are no monosaccharides in this entry.

6.4 Ligands (i)

In the following table, the Atoms column lists the number of modelled atoms in the group and the number defined in the chemical component dictionary. The B-factors column lists the minimum, median, 95th percentile and maximum values of B factors of atoms in the group. The column labelled 'Q< 0.9' lists the number of atoms with occupancy less than 0.9.

Mol	Type	Chain	Res	Atoms	RSCC	RSR	B-factors(Å^2)	Q<0.9
4	GOL	B	805	6/6	0.61	0.17	48,51,55,59	0
4	GOL	A	808	6/6	0.78	0.27	42,45,46,48	0
3	SO4	B	803	5/5	0.85	0.34	43,51,68,82	0
3	SO4	B	804	5/5	0.86	0.14	72,75,82,97	0
3	SO4	A	807	5/5	0.86	0.32	46,47,73,74	0
3	SO4	A	806	5/5	0.86	0.27	52,54,69,94	0
2	UKI	B	801	34/34	0.94	0.14	20,28,72,87	0
2	UKI	A	801	34/34	0.95	0.11	16,24,46,48	0
3	SO4	A	805	5/5	0.95	0.35	72,82,85,96	0
3	SO4	B	802	5/5	0.97	0.14	44,52,56,75	0
3	SO4	A	803	5/5	0.98	0.07	33,34,39,42	0
3	SO4	A	804	5/5	0.98	0.10	36,43,47,50	0
3	SO4	A	802	5/5	0.99	0.08	31,33,36,50	0

The following is a graphical depiction of the model fit to experimental electron density of all instances of the Ligand of Interest. In addition, ligands with molecular weight > 250 and outliers as shown on the geometry validation Tables will also be included. Each fit is shown from different orientation to approximate a three-dimensional view.

For Manuscript Review

Electron density around UKI B 801:

$2mF_o$-DF_c (at 0.7 rmsd) in gray
mF_o-DF_c (at 3 rmsd) in purple (negative)
and green (positive)

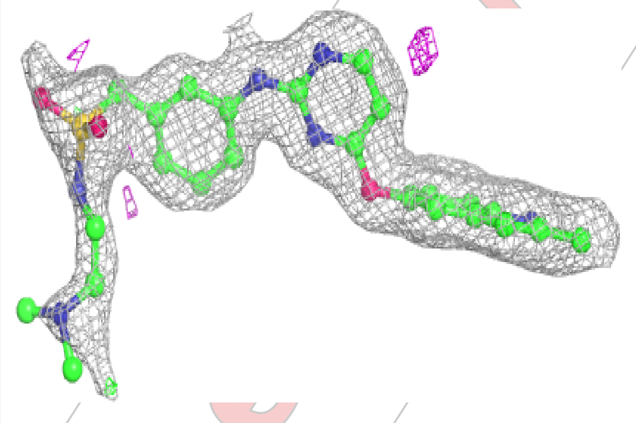

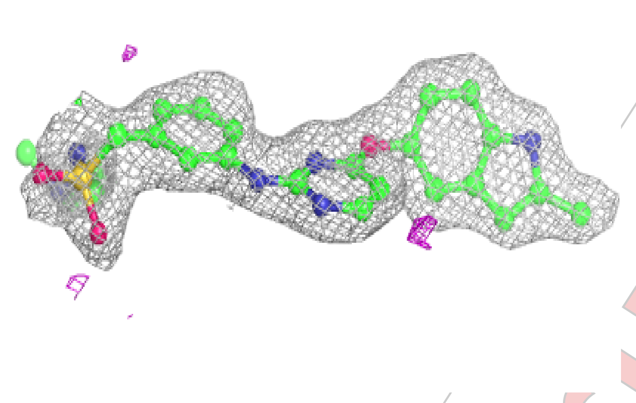

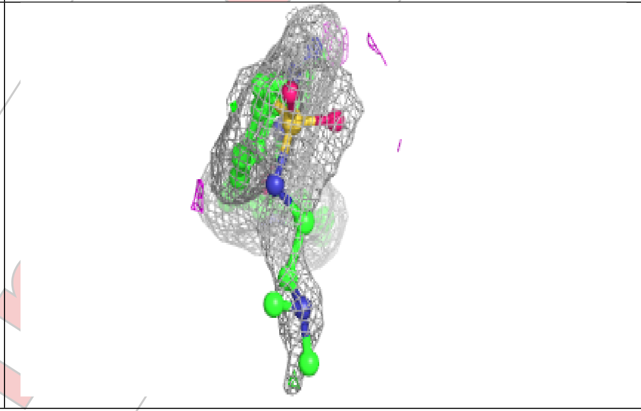

Electron density around UKI A 801:

$2mF_o$-DF_c (at 0.7 rmsd) in gray
mF_o-DF_c (at 3 rmsd) in purple (negative)
and green (positive)

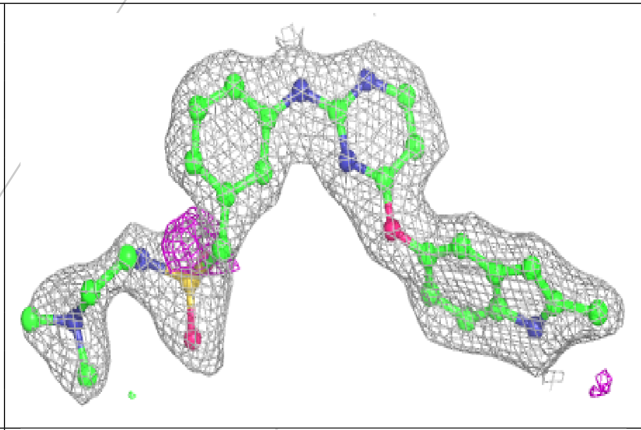

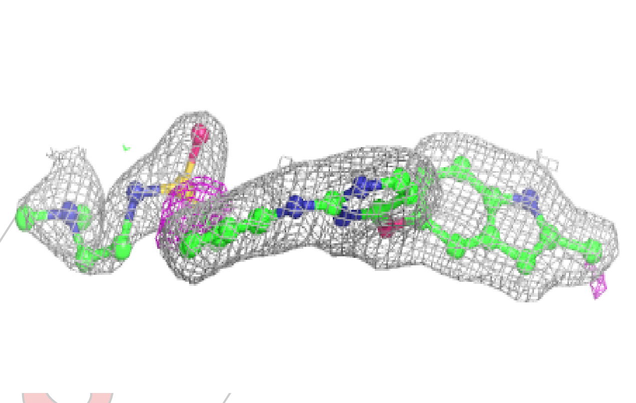

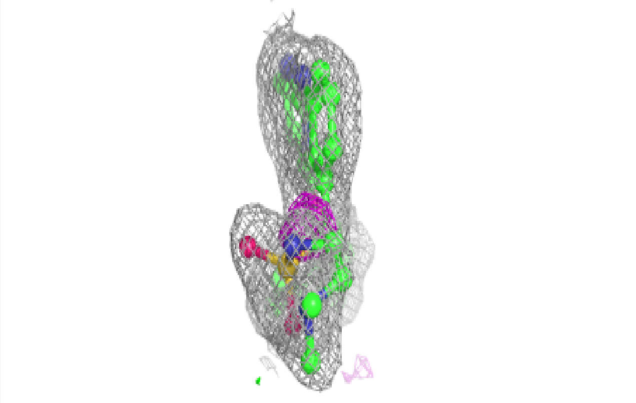

6.5 Other polymers (i)

There are no such residues in this entry.

For Manuscript Review
