# Supplementary material for: Structural basis and selectivity of sulfatinib binding to FGFR and CSF-1R
Source: Commun Chem. 2024 Jan 3;7:3. doi: 10.1038/s42004-023-01084-0 (PMC10764862; doi:10.1038/s42004-023-01084-0)
Supplement: Supplementary file 4 — Supplementary Data 2 [file 42004_2023_1084_MOESM4_ESM.zip › supplementary data 2/sulfatinib_CSF-1R_val-report-full-annotate_P1.pdf]

Full wwPDB X-ray Structure Validation Report (i)

Jun 15, 2023 – 05:38 PM JST

PDB ID	:	8JOT
Title	:	Crystal structure of CSF-1R kinase domain with sulfatinib
Deposited on	:	2023-06-08
Resolution	:	1.69 Å(reported)

This wwPDB validation report is for manuscript review

For Manuscript Review

This is a Full wwPDB X-ray Structure Validation Report.

This report is produced by the wwPDB biocuration pipeline after annotation of the structure.

We welcome your comments at *validation@mail.wwpdb.org*
A user guide is available at
https://www.wwpdb.org/validation/2017/XrayValidationReportHelp
with specific help available everywhere you see the (i) symbol.

The types of validation reports are described at
http://www.wwpdb.org/validation/2017/FAQs#types.

The following versions of software and data (see references (i)) were used in the production of this report:

MolProbity	:	4.02b-467
Mogul	:	1.8.5 (274361), CSD as541be (2020)
Xtriage (Phenix)	:	1.13
EDS	:	2.33
buster-report	:	1.1.7 (2018)
Percentile statistics	:	20191225.v01 (using entries in the PDB archive December 25th 2019)
Refmac	:	5.8.0158
CCP4	:	7.0.044 (Gargrove)
Ideal geometry (proteins)	:	Engh & Huber (2001)
Ideal geometry (DNA, RNA)	:	Parkinson et al. (1996)

1 Overall quality at a glance (i)

The following experimental techniques were used to determine the structure:
X-RAY DIFFRACTION

The reported resolution of this entry is 1.69 Å.

Percentile scores (ranging between 0-100) for global validation metrics of the entry are shown in the following graphic. The table shows the number of entries on which the scores are based.

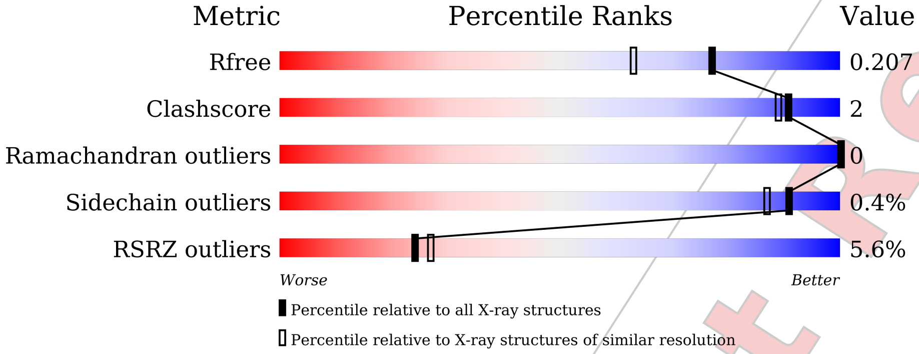

Metric	Whole archive (#Entries)	Similar resolution (#Entries, resolution range(Å))
R_{free}	130704	4298 (1.70-1.70)
Clashscore	141614	4695 (1.70-1.70)
Ramachandran outliers	138981	4610 (1.70-1.70)
Sidechain outliers	138945	4610 (1.70-1.70)
RSRZ outliers	127900	4222 (1.70-1.70)

The table below summarises the geometric issues observed across the polymeric chains and their fit to the electron density. The red, orange, yellow and green segments of the lower bar indicate the fraction of residues that contain outliers for >=3, 2, 1 and 0 types of geometric quality criteria respectively. A grey segment represents the fraction of residues that are not modelled. The numeric value for each fraction is indicated below the corresponding segment, with a dot representing fractions <=5% The upper red bar (where present) indicates the fraction of residues that have poor fit to the electron density. The numeric value is given above the bar.

Mol	Chain	Length	Quality of chain
1	A	354	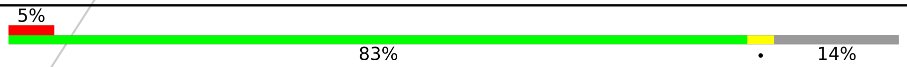

Validation Pipeline (wwPDB-VP) : 2.33

For Manuscript Review

2 Entry composition (i)

There are 4 unique types of molecules in this entry. The entry contains 2722 atoms, of which 0 are hydrogens and 0 are deuteriums.

In the tables below, the ZeroOcc column contains the number of atoms modelled with zero occupancy, the AltConf column contains the number of residues with at least one atom in alternate conformation and the Trace column contains the number of residues modelled with at most 2 atoms.

- Molecule 1 is a protein called Macrophage colony-stimulating factor 1 receptor.

Mol	Chain	Residues	Atoms					ZeroOcc	AltConf	Trace
			Total	C	N	O	S			
1	A	306	2370	1534	400	423	13	0	0	0

There are 49 discrepancies between the modelled and reference sequences:

Chain	Residue	Modelled	Actual	Comment	Reference
A	531	MET	-	initiating methionine	UNP P07333
A	532	LYS	-	expression tag	UNP P07333
A	533	LYS	-	expression tag	UNP P07333
A	534	GLY	-	expression tag	UNP P07333
A	535	HIS	-	expression tag	UNP P07333
A	536	HIS	-	expression tag	UNP P07333
A	537	HIS	-	expression tag	UNP P07333
A	538	HIS	-	expression tag	UNP P07333
A	539	HIS	-	expression tag	UNP P07333
A	540	HIS	-	expression tag	UNP P07333
A	541	GLY	-	expression tag	UNP P07333
A	667	THR	CYS	engineered mutation	UNP P07333
A	?	-	LYS	deletion	UNP P07333
A	?	-	TYR	deletion	UNP P07333
A	?	-	VAL	deletion	UNP P07333
A	?	-	ARG	deletion	UNP P07333
A	?	-	ARG	deletion	UNP P07333
A	?	-	ASP	deletion	UNP P07333
A	?	-	SER	deletion	UNP P07333
A	?	-	GLY	deletion	UNP P07333
A	?	-	PHE	deletion	UNP P07333
A	?	-	SER	deletion	UNP P07333
A	?	-	SER	deletion	UNP P07333
A	?	-	GLN	deletion	UNP P07333
A	?	-	GLY	deletion	UNP P07333
A	?	-	VAL	deletion	UNP P07333
A	?	-	ASP	deletion	UNP P07333

Continued on next page...

For Manuscript Review

Continued from previous page...

Chain	Residue	Modelled	Actual	Comment	Reference
A	?	-	THR	deletion	UNP P07333
A	?	-	TYR	deletion	UNP P07333
A	?	-	VAL	deletion	UNP P07333
A	?	-	GLU	deletion	UNP P07333
A	?	-	MET	deletion	UNP P07333
A	?	-	ARG	deletion	UNP P07333
A	?	-	PRO	deletion	UNP P07333
A	?	-	VAL	deletion	UNP P07333
A	?	-	SER	deletion	UNP P07333
A	?	-	THR	deletion	UNP P07333
A	?	-	SER	deletion	UNP P07333
A	?	-	SER	deletion	UNP P07333
A	?	-	ASN	deletion	UNP P07333
A	?	-	ASP	deletion	UNP P07333
A	?	-	SER	deletion	UNP P07333
A	?	-	PHE	deletion	UNP P07333
A	?	-	SER	deletion	UNP P07333
A	?	-	GLU	deletion	UNP P07333
A	?	-	GLN	deletion	UNP P07333
A	?	-	ASP	deletion	UNP P07333
A	830	SER	CYS	engineered mutation	UNP P07333
A	907	THR	CYS	engineered mutation	UNP P07333

- Molecule 2 is N-(2-(dimethylamino)ethyl)-1-(3-((4-((2-methyl-1H-indol-5-yl)oxy)pyrimidin -2-yl)amino)phenyl)methanesulfonamide (three-letter code: UKI) (formula: $C_{24}H_{28}N_6O_3S$) (labeled as "Ligand of Interest" by depositor).

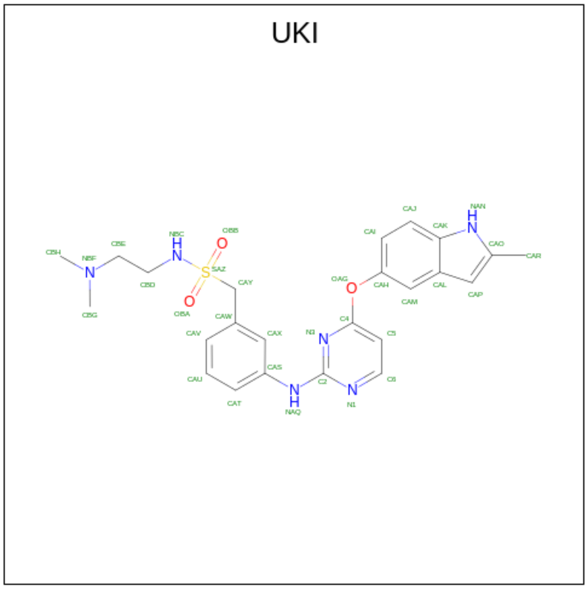

For Manuscript Review

Mol	Chain	Residues	Atoms					ZeroOcc	AltConf
			Total	C	N	O	S		
2	A	1	34	24	6	3	1	0	0

- Molecule 3 is GLYCEROL (three-letter code: GOL) (formula: $C_3H_8O_3$).

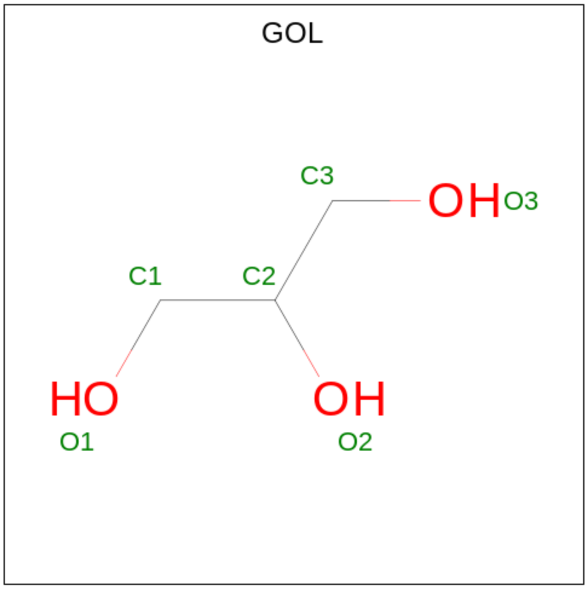

Mol	Chain	Residues	Atoms			ZeroOcc	AltConf
			Total	C	O		
3	A	1	6	3	3	0	0

- Molecule 4 is water.

Mol	Chain	Residues	Atoms		ZeroOcc	AltConf
			Total	O		
4	A	312	312	312	0	0

For Manuscript Review

3 Residue-property plots (i)

These plots are drawn for all protein, RNA, DNA and oligosaccharide chains in the entry. The first graphic for a chain summarises the proportions of the various outlier classes displayed in the second graphic. The second graphic shows the sequence view annotated by issues in geometry and electron density. Residues are color-coded according to the number of geometric quality criteria for which they contain at least one outlier: green = 0, yellow = 1, orange = 2 and red = 3 or more. A red dot above a residue indicates a poor fit to the electron density (RSRZ > 2). Stretches of 2 or more consecutive residues without any outlier are shown as a green connector. Residues present in the sample, but not in the model, are shown in grey.

- Molecule 1: Macrophage colony-stimulating factor 1 receptor

Chain A:
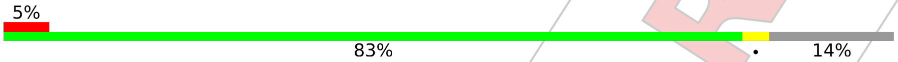

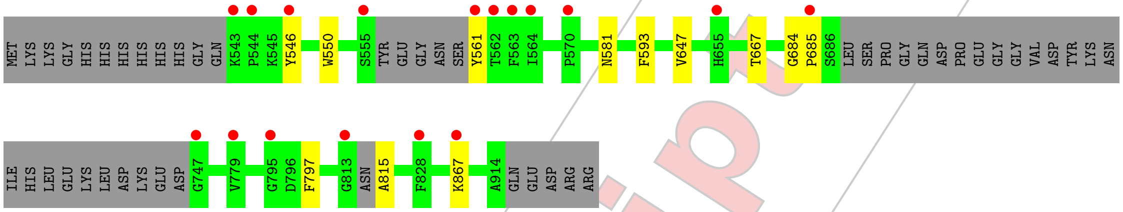

For Manuscript Review

4 Data and refinement statistics (i)

Property	Value	Source
Space group	P 43 21 2	Depositor
Cell constants<br>a, b, c, α, β, γ	64.31Å 64.31Å 184.69Å<br>90.00° 90.00° 90.00°	Depositor
Resolution (Å)	36.58 – 1.69<br>36.58 – 1.69	Depositor<br>EDS
% Data completeness<br>(in resolution range)	99.6 (36.58-1.69)<br>99.6 (36.58-1.69)	Depositor<br>EDS
R_{merge}	(Not available)	Depositor
R_{sym}	(Not available)	Depositor
$< I/\sigma(I) >$ [1]	1.56 (at 1.69Å)	Xtriage
Refinement program	PHENIX (1.19.2_4158: ???)	Depositor
R, R_{free}	0.179 , 0.207<br>0.180 , 0.207	Depositor<br>DCC
R_{free} test set	1998 reflections (4.51%)	wwPDB-VP
Wilson B-factor (Å^2)	23.6	Xtriage
Anisotropy	0.327	Xtriage
Bulk solvent k_{sol}(e/Å^3), B_{sol}(Å^2)	0.37 , 53.5	EDS
L-test for twinning[2]	$< \|L\| > = 0.49$, $< L^2 > = 0.32$	Xtriage
Estimated twinning fraction	No twinning to report.	Xtriage
F_o,F_c correlation	0.97	EDS
Total number of atoms	2722	wwPDB-VP
Average B, all atoms (Å^2)	30.0	wwPDB-VP

Xtriage's analysis on translational NCS is as follows: *The largest off-origin peak in the Patterson function is 5.72% of the height of the origin peak. No significant pseudotranslation is detected.*

[1] Intensities estimated from amplitudes.
[2] Theoretical values of $< |L| >$, $< L^2 >$ for acentric reflections are 0.5, 0.333 respectively for untwinned datasets, and 0.375, 0.2 for perfectly twinned datasets.

For Manuscript Review

5 Model quality (i)

5.1 Standard geometry (i)

Bond lengths and bond angles in the following residue types are not validated in this section: GOL, UKI

The Z score for a bond length (or angle) is the number of standard deviations the observed value is removed from the expected value. A bond length (or angle) with $|Z| > 5$ is considered an outlier worth inspection. RMSZ is the root-mean-square of all Z scores of the bond lengths (or angles).

Mol	Chain	Bond lengths		Bond angles	
		RMSZ	#$\|Z\|$ >5	RMSZ	#$\|Z\|$ >5
1	A	0.36	0/2428	0.56	0/3295

There are no bond length outliers.

There are no bond angle outliers.

There are no chirality outliers.

There are no planarity outliers.

5.2 Too-close contacts (i)

In the following table, the Non-H and H(model) columns list the number of non-hydrogen atoms and hydrogen atoms in the chain respectively. The H(added) column lists the number of hydrogen atoms added and optimized by MolProbity. The Clashes column lists the number of clashes within the asymmetric unit, whereas Symm-Clashes lists symmetry-related clashes.

Mol	Chain	Non-H	H(model)	H(added)	Clashes	Symm-Clashes
1	A	2370	0	2329	8	0
2	A	34	0	0	0	0
3	A	6	0	8	1	0
4	A	312	0	0	4	0
All	All	2722	0	2337	8	0

The all-atom clashscore is defined as the number of clashes found per 1000 atoms (including hydrogen atoms). The all-atom clashscore for this structure is 2.

All (8) close contacts within the same asymmetric unit are listed below, sorted by their clash magnitude.

For Manuscript Review

Atom-1	Atom-2	Interatomic distance (Å)	Clash overlap (Å)
1:A:815:ALA:N	4:A:1103:HOH:O	2.23	0.72
1:A:561:TYR:N	4:A:1106:HOH:O	2.29	0.65
1:A:667:THR:HG23	4:A:1104:HOH:O	2.04	0.56
1:A:647:VAL:HG21	1:A:797:PHE:HZ	1.73	0.53
1:A:581:ASN:ND2	4:A:1107:HOH:O	2.33	0.49
1:A:546:TYR:HB2	1:A:593:PHE:CG	2.53	0.44
1:A:550:TRP:HB2	3:A:1002:GOL:H11	2.01	0.42
1:A:684:GLY:N	1:A:685:PRO:HD2	2.35	0.42

There are no symmetry-related clashes.

5.3 Torsion angles (i)

5.3.1 Protein backbone (i)

In the following table, the Percentiles column shows the percent Ramachandran outliers of the chain as a percentile score with respect to all X-ray entries followed by that with respect to entries of similar resolution.

The Analysed column shows the number of residues for which the backbone conformation was analysed, and the total number of residues.

Mol	Chain	Analysed	Favoured	Allowed	Outliers	Percentiles	
1	A	298/354 (84%)	295 (99%)	3 (1%)	0	100	100

There are no Ramachandran outliers to report.

5.3.2 Protein sidechains (i)

In the following table, the Percentiles column shows the percent sidechain outliers of the chain as a percentile score with respect to all X-ray entries followed by that with respect to entries of similar resolution.

The Analysed column shows the number of residues for which the sidechain conformation was analysed, and the total number of residues.

Mol	Chain	Analysed	Rotameric	Outliers	Percentiles	
1	A	245/299 (82%)	244 (100%)	1 (0%)	91	87

All (1) residues with a non-rotameric sidechain are listed below:

For Manuscript Review

Mol	Chain	Res	Type
1	A	867	LYS

Sometimes sidechains can be flipped to improve hydrogen bonding and reduce clashes. There are no such sidechains identified.

5.3.3 RNA (i)

There are no RNA molecules in this entry.

5.4 Non-standard residues in protein, DNA, RNA chains (i)

There are no non-standard protein/DNA/RNA residues in this entry.

5.5 Carbohydrates (i)

There are no monosaccharides in this entry.

5.6 Ligand geometry (i)

2 ligands are modelled in this entry.

In the following table, the Counts columns list the number of bonds (or angles) for which Mogul statistics could be retrieved, the number of bonds (or angles) that are observed in the model and the number of bonds (or angles) that are defined in the Chemical Component Dictionary. The Link column lists molecule types, if any, to which the group is linked. The Z score for a bond length (or angle) is the number of standard deviations the observed value is removed from the expected value. A bond length (or angle) with $|Z| > 2$ is considered an outlier worth inspection. RMSZ is the root-mean-square of all Z scores of the bond lengths (or angles).

Mol	Type	Chain	Res	Link	Bond lengths			Bond angles		
					Counts	RMSZ	$\#\|Z\| > 2$	Counts	RMSZ	$\#\|Z\| > 2$
2	UKI	A	1001	-	34,37,37	2.48	9 (26%)	45,52,52	2.44	10 (22%)
3	GOL	A	1002	-	5,5,5	0.72	0	5,5,5	1.05	0

In the following table, the Chirals column lists the number of chiral outliers, the number of chiral centers analysed, the number of these observed in the model and the number defined in the Chemical Component Dictionary. Similar counts are reported in the Torsion and Rings columns. '-' means no outliers of that kind were identified.

Mol	Type	Chain	Res	Link	Chirals	Torsions	Rings
2	UKI	A	1001	-	-	5/20/20/20	0/4/4/4

Continued on next page...

For Manuscript Review

Continued from previous page...

Mol	Type	Chain	Res	Link	Chirals	Torsions	Rings
3	GOL	A	1002	-	-	0/4/4/4	-

All (9) bond length outliers are listed below:

Mol	Chain	Res	Type	Atoms	Z	Observed(Å)	Ideal(Å)
2	A	1001	UKI	OBB-SAZ	7.58	1.54	1.43
2	A	1001	UKI	CAY-CAW	-6.15	1.40	1.50
2	A	1001	UKI	OBA-SAZ	5.88	1.51	1.43
2	A	1001	UKI	CAJ-CAK	-4.31	1.34	1.41
2	A	1001	UKI	CAP-CAO	-4.27	1.33	1.39
2	A	1001	UKI	CAM-CAL	-3.22	1.34	1.42
2	A	1001	UKI	C6-N1	2.50	1.39	1.34
2	A	1001	UKI	CAS-NAQ	-2.34	1.35	1.40
2	A	1001	UKI	CAM-CAH	2.12	1.40	1.37

All (10) bond angle outliers are listed below:

Mol	Chain	Res	Type	Atoms	Z	Observed(^{o})	Ideal(^{o})
2	A	1001	UKI	OBB-SAZ-OBA	-7.66	108.26	119.35
2	A	1001	UKI	N1-C2-N3	-6.89	120.03	126.55
2	A	1001	UKI	C6-N1-C2	6.27	121.01	115.45
2	A	1001	UKI	C2-N3-C4	5.48	121.43	114.99
2	A	1001	UKI	CBD-NBC-SAZ	-4.41	109.27	122.55
2	A	1001	UKI	C5-C6-N1	-4.40	118.50	123.96
2	A	1001	UKI	CAR-CAO-NAN	2.23	125.15	119.98
2	A	1001	UKI	CAI-CAH-CAM	-2.23	117.81	120.81
2	A	1001	UKI	NAQ-C2-N3	2.12	124.13	116.92
2	A	1001	UKI	OAG-C4-C5	2.04	119.44	115.35

There are no chirality outliers.

All (5) torsion outliers are listed below:

Mol	Chain	Res	Type	Atoms
2	A	1001	UKI	CBD-CBE-NBF-CBG
2	A	1001	UKI	NBC-CBD-CBE-NBF
2	A	1001	UKI	CBD-NBC-SAZ-OBB
2	A	1001	UKI	CBD-CBE-NBF-CBH
2	A	1001	UKI	CBD-NBC-SAZ-CAY

There are no ring outliers.

1 monomer is involved in 1 short contact:

For Manuscript Review

Mol	Chain	Res	Type	Clashes	Symm-Clashes
3	A	1002	GOL	1	0

The following is a two-dimensional graphical depiction of Mogul quality analysis of bond lengths, bond angles, torsion angles, and ring geometry for all instances of the Ligand of Interest. In addition, ligands with molecular weight > 250 and outliers as shown on the validation Tables will also be included. For torsion angles, if less then 5% of the Mogul distribution of torsion angles is within 10 degrees of the torsion angle in question, then that torsion angle is considered an outlier. Any bond that is central to one or more torsion angles identified as an outlier by Mogul will be highlighted in the graph. For rings, the root-mean-square deviation (RMSD) between the ring in question and similar rings identified by Mogul is calculated over all ring torsion angles. If the average RMSD is greater than 60 degrees and the minimal RMSD between the ring in question and any Mogul-identified rings is also greater than 60 degrees, then that ring is considered an outlier. The outliers are highlighted in purple. The color gray indicates Mogul did not find sufficient equivalents in the CSD to analyse the geometry.

Ligand UKI A 1001

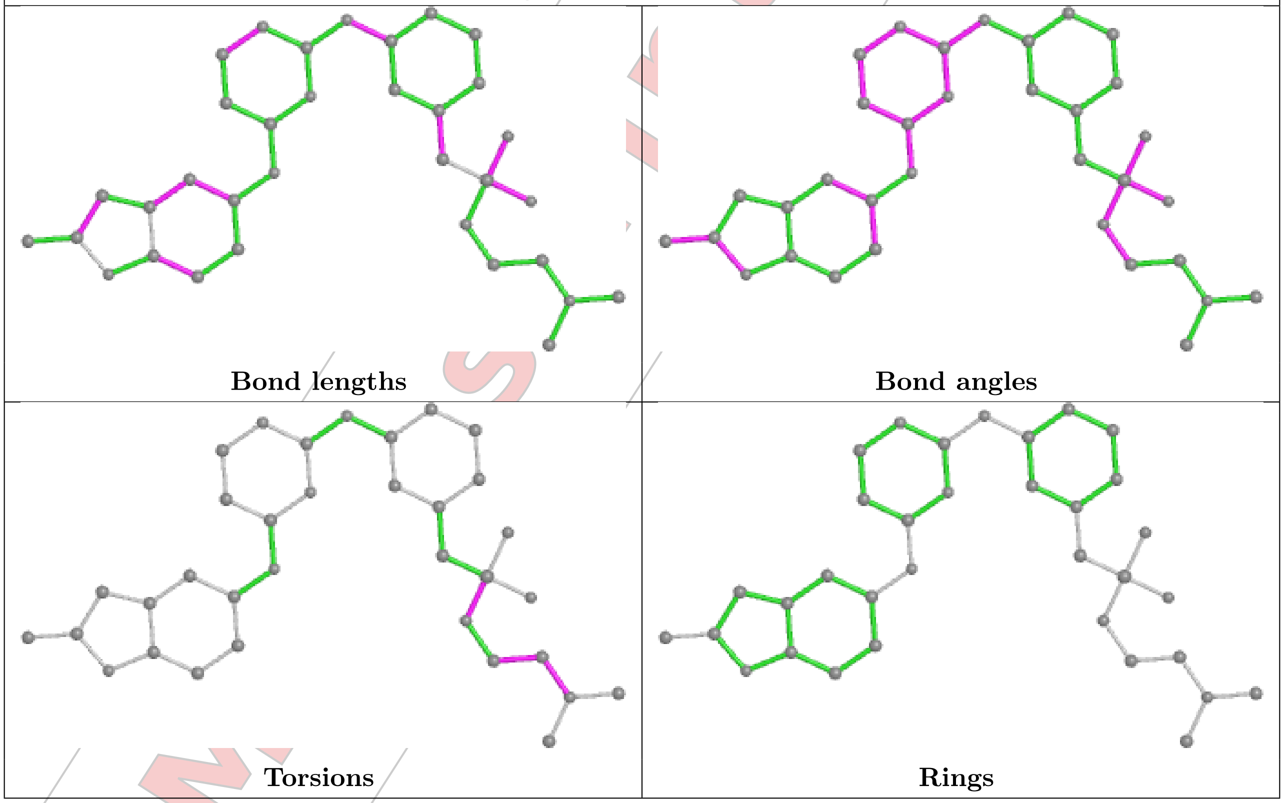

5.7 Other polymers (i)

There are no such residues in this entry.

For Manuscript Review

5.8 Polymer linkage issues (i)

There are no chain breaks in this entry.

For Manuscript Review

6 Fit of model and data (i)

6.1 Protein, DNA and RNA chains (i)

In the following table, the column labelled '#RSRZ> 2' contains the number (and percentage) of RSRZ outliers, followed by percent RSRZ outliers for the chain as percentile scores relative to all X-ray entries and entries of similar resolution. The OWAB column contains the minimum, median, 95th percentile and maximum values of the occupancy-weighted average B-factor per residue. The column labelled 'Q< 0.9' lists the number of (and percentage) of residues with an average occupancy less than 0.9.

Mol	Chain	Analysed	<RSRZ>	#RSRZ>2			OWAB(Å^2)	Q<0.9
1	A	306/354 (86%)	0.07	17 (5%)	24	27	17, 26, 48, 75	0

All (17) RSRZ outliers are listed below:

Mol	Chain	Res	Type	RSRZ
1	A	561	TYR	6.4
1	A	813	GLY	4.0
1	A	747	GLY	3.8
1	A	555	SER	3.5
1	A	570	PRO	3.1
1	A	779	VAL	3.0
1	A	544	PRO	2.9
1	A	685	PRO	2.9
1	A	563	PHE	2.9
1	A	564	ILE	2.8
1	A	828	PHE	2.8
1	A	867	LYS	2.6
1	A	543	LYS	2.4
1	A	655	HIS	2.4
1	A	795	GLY	2.2
1	A	546	TYR	2.1
1	A	562	THR	2.1

6.2 Non-standard residues in protein, DNA, RNA chains (i)

There are no non-standard protein/DNA/RNA residues in this entry.

6.3 Carbohydrates (i)

There are no monosaccharides in this entry.

For Manuscript Review

6.4 Ligands (i)

In the following table, the Atoms column lists the number of modelled atoms in the group and the number defined in the chemical component dictionary. The B-factors column lists the minimum, median, 95th percentile and maximum values of B factors of atoms in the group. The column labelled 'Q< 0.9' lists the number of atoms with occupancy less than 0.9.

Mol	Type	Chain	Res	Atoms	RSCC	RSR	B-factors(Å^2)	Q<0.9
3	GOL	A	1002	6/6	0.86	0.15	36,41,49,55	0
2	UKI	A	1001	34/34	0.91	0.13	17,26,83,88	0

The following is a graphical depiction of the model fit to experimental electron density of all instances of the Ligand of Interest. In addition, ligands with molecular weight > 250 and outliers as shown on the geometry validation Tables will also be included. Each fit is shown from different orientation to approximate a three-dimensional view.

Electron density around UKI A 1001:

2mF$_o$-DF$_c$ (at 0.7 rmsd) in gray
mF$_o$-DF$_c$ (at 3 rmsd) in purple (negative)
and green (positive)

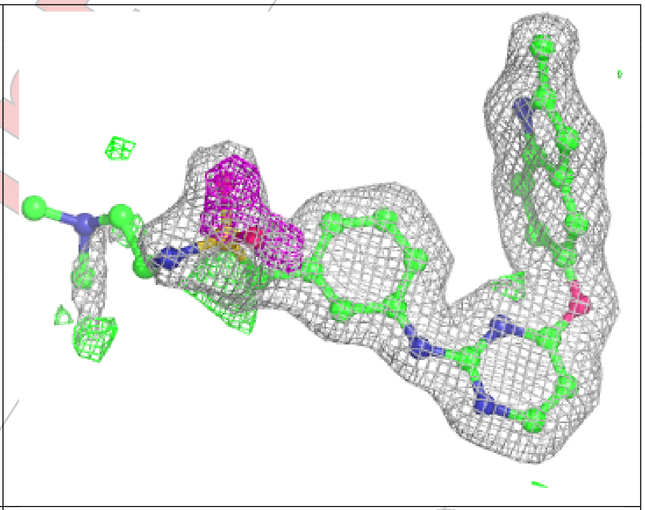

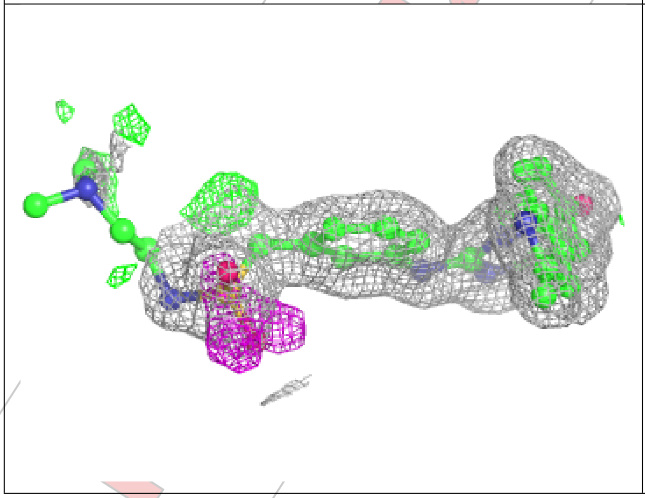

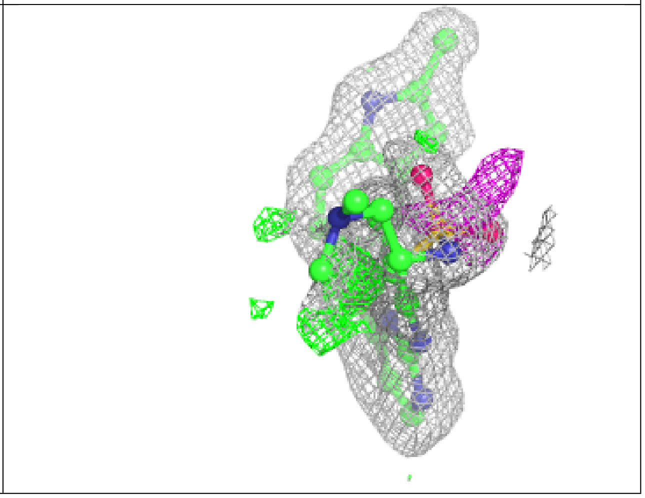

6.5 Other polymers (i)

There are no such residues in this entry.

For Manuscript Review
